# Macrophages directly contribute collagen to scar formation during zebrafish heart regeneration and mouse heart repair

**DOI:** 10.1038/s41467-019-14263-2

**Published:** 2020-01-30

**Authors:** Filipa C. Simões, Thomas J. Cahill, Amy Kenyon, Daria Gavriouchkina, Joaquim M. Vieira, Xin Sun, Daniela Pezzolla, Christophe Ravaud, Eva Masmanian, Michael Weinberger, Sarah Mayes, Madeleine E. Lemieux, Damien N. Barnette, Mala Gunadasa-Rohling, Ruth M. Williams, David R. Greaves, Le A. Trinh, Scott E. Fraser, Sarah L. Dallas, Robin P. Choudhury, Tatjana Sauka-Spengler, Paul R. Riley

**Affiliations:** 1 Department of Physiology, Anatomy and Genetics, University of Oxford, Oxford, OX1 3PT UK; 2 Radcliffe Department of Medicine, MRC Weatherall Institute of Molecular Medicine, University of Oxford, Oxford, OX3 9DS UK; 3 BHF Oxbridge Centre of Regenerative Medicine, University of Oxford, Oxford, UK; 4 Division of Cardiovascular Medicine, Radcliffe Department of Medicine, University of Oxford, Oxford, UK; 5 Bioinfo, Plantagenet, Canada; 6 Sir William Dunn School of Pathology, University of Oxford, Oxford, UK; 7 Translational Imaging Centre, University of Southern California, Los Angeles, CA USA; 8 School of Dentistry, University of Missouri-Kansas City, Kansas City, MO USA; 9 Present Address: Molecular Genetics Unit, Okinawa Institute of Science & Technology, 1919-1 Tancha, Onna, 904-0495 Japan

**Keywords:** Innate immune cells, Cardiac regeneration

## Abstract

Canonical roles for macrophages in mediating the fibrotic response after a heart attack include extracellular matrix turnover and activation of cardiac fibroblasts to initiate collagen deposition. Here we reveal that macrophages directly contribute collagen to the forming post-injury scar. Unbiased transcriptomics shows an upregulation of collagens in both zebrafish and mouse macrophages following heart injury. Adoptive transfer of macrophages, from either collagen-tagged zebrafish or adult mouse GFP*tpz*-collagen donors, enhances scar formation via cell autonomous production of collagen. In zebrafish, the majority of tagged collagen localises proximal to the injury, within the overlying epicardial region, suggesting a possible distinction between macrophage-deposited collagen and that predominantly laid-down by myofibroblasts. Macrophage-specific targeting of *col4a3bpa* and cognate *col4a1* in zebrafish significantly reduces scarring in cryoinjured hosts. Our findings contrast with the current model of scarring, whereby collagen deposition is exclusively attributed to myofibroblasts, and implicate macrophages as direct contributors to fibrosis during heart repair.

## Abstract (web summary)

Macrophages mediate the fibrotic response after a heart attack by extracellular matrix turnover and cardiac fibroblasts activation. Here the authors identify an evolutionarily-conserved function of macrophages that contributes directly to the forming post-injury scar through cell-autonomous deposition of collagen.

## Introduction

Macrophages are highly plastic and functionally diverse during steady-state conditions and following tissue injury or disease (reviewed in ref. ^1^). In the heart, tissue-resident macrophages have been implicated in coronary vessel development^2^ and more recently in contributing to cardiac conduction^3^. After myocardial infarction (MI), macrophages are the predominant immune cell type; they arise from monocytes which infiltrate from the spleen and bone marrow^4^ and function through context-dependent actions (reviewed in ref. ^5^). During the initial inflammatory phase, macrophages adopt a classically activated-like state, associated with the release of pro-inflammatory cytokines, and function to digest and remove dying neutrophils, dead tissue and necrotic debris by releasing proteolytic enzymes and reactive oxygen species. Subsequently, they transition to a reparative state and contribute to fibrosis via the production of cytokines, chemokines and growth factors, such as TGFβ and PDGF. These alternatively-activated macrophages modify extracellular matrix (ECM) turnover by regulating the balance of matrix metalloproteinases (MMPs) and their tissue inhibitors, and activate cardiac fibroblasts to become myofibroblasts, which deposit collagen to form a scar. Ultimately, the replacement of dead cardiomyocytes with non-contractile scar tissue stabilises the injury site but leads to longer-term pathological ventricular remodelling and heart failure.

In contrast to the situation in humans, several species are capable of regenerating the injured heart, either in the absence of scar formation or via scar resolution. In the zebrafish, resection of the ventricle results in complete heart regeneration, without stable scar formation. The post-resection zebrafish heart differs in many ways to the infarcted human heart, as it lacks ischemia-induced cell death and forms a fibril layer, mainly composed of fibrin fibres with only minor collagen deposition^6^. In contrast, following cryoinjury, heart regeneration is delayed, coincident with massive cell death and fibrotic scar formation. This, in turn, resembles more closely the human heart post-MI; however, following cryoinjury, regeneration occurs even in the presence of scarring^7,8^. In neonatal mice the heart can regenerate during a limited temporal window, which extends to ~7 days after birth, following either MI or ventricular resection^9^.

The transition from a tissue repair phase to a regenerative phase following heart injury requires an orchestrated and balanced immune response to fine-tune the interplay between a pro-fibrotic and a pro-regenerative environment^10–15^. Macrophages are integral to both repair by scar formation and tissue regeneration^10–16^; however, factors that drive them towards either a regenerative or pro-fibrotic phenotype are largely unknown. Here we analyse the macrophage responses during resection versus cryoinjury in zebrafish and in neonatal versus adult conditions of heart repair in mice. We identify an evolutionarily-conserved function of macrophages that contributes directly to the forming scar through cell-autonomous deposition of collagen. Thus, we add significantly to the functional repertoire attributed to macrophages and challenge the prevailing view that myofibroblasts alone drive fibrosis and scar formation in the injured heart.

## Results

### Spatiotemporal macrophage responses to cardiac injury

We first determined the temporal and dynamic response of macrophages to injury in both adult zebrafish^6–8^ and neonatal versus adult mouse models. In zebrafish, we performed multiplex Nanostring nCounter gene expression analysis at 11 time points following either resection or cryoinjury (1 hour post-injury (hpi), 3 hpi, 5 hpi, 1 day post-injury (dpi), 3 dpi, 4 dpi, 5 dpi, 6 dpi, 8 dpi, 10 dpi and 14 dpi; Supplementary Fig. 1a, b). The macrophage-specific marker *mpeg1* revealed increased infiltration at 1 dpi in both models, which was accompanied by neutrophil infiltration as determined by tracking *mpx* expression. Whereas neutrophil presence at the site of injury was short-lived, macrophages persisted for an extended period in both injury settings (Supplementary Fig. 1b). This profile was supported by expression of the pan-leukocytic marker *l-plastin* and inflammatory markers *il8* and *il1*β (Supplementary Fig. 1b). In contrast, the pro-resolving marker, *tgfβ* exhibited a dynamic pattern of expression from 1 hpi to 14 dpi (Supplementary Fig. 1b). We next determined the spatial distribution of macrophages at 1 dpi when *mpeg1* expression first increased after cardiac injury, and at 5 dpi, when *mpeg1* was significantly higher upon cryoinjury (as compared to ventricle resection; Supplementary Fig. 1c–q). Analysis of mpeg1-stained sections showed macrophages distributed in the atrium, throughout the ventricle and in close proximity to the epicardium, with increased incidence more proximal to the site of injury (Supplementary Fig. 1c–q). By 14 dpi, we observed resolution of inflammation (fewer macrophages) following ventricular resection, while mpeg1 expression was still high following cryoinjury and subsequent scarring (Supplementary Fig. 1r–u).

In the mouse, we invoked MI in P1, P7 and adult stages (Supplementary Fig. 2a–c), and observed CD68^+^ macrophages localised to the infarct zone at day 4 (Supplementary Fig. 2d). We directly quantified the number of macrophages in the neonatal and adult responses by flow cytometry (CD45^+^ CD11b^+^ Ly6G^−^ F4/80^+^), as compared with leucocytes (CD45^+^), myeloid cells (CD45^+^ CD11b^+^), neutrophils (CD45^+^ CD11b^+^ Ly6G^+^) and monocytes (CD45^+^ CD11b^+^ Ly6G^−^ F4/80^−^Ly6C^hi/lo^)^17^ (Supplementary Fig. 2e–n). Relative to the neonatal response, leucocytes, myeloid cells and specifically CD45^+^ CD11b^+^ Ly6G^−^ F4/80^+^ macrophages were significantly elevated in adult infarcted hearts across days 1 and 4 (percentage of live cells: adult 12.86 ± 2.021, *n* = 5 vs P7 6.205 ± 0.446, *n* = 4 vs P1 5.623 ± 1.142, *n* = 6, **p *=* 0.006, data are mean ± SEM, one-way ANOVA). By day 7, the numbers of all immune cell types were reduced to baseline across all three stages. Alternatively activated, CD206-positive ^18^ macrophages were significantly increased in adult hearts relative to P1 or P7 at days 4 and 7, consistent with a significantly dampened macrophage response in neonates compared to adults (Supplementary Fig. 2o, p).

### Transcriptional differences in the macrophage responses during regeneration and scar formation

To gain insight into the molecular programmes driving the macrophage response during transient scarring and regeneration as opposed to stable scar formation, we compared macrophage transcriptomes in adult zebrafish following resection versus cryoinjury and in post-MI mice, at neonatal P1 and P7 stages versus the adult.

**Fig. 1:**
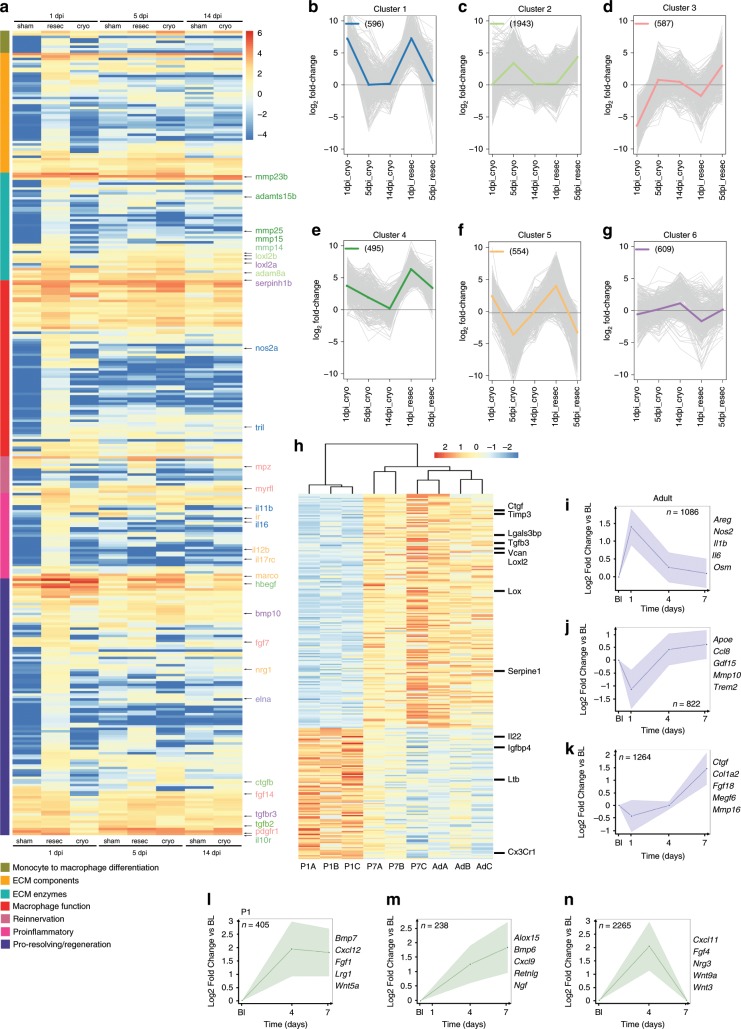
Transcriptomic analysis of macrophages in zebrafish and mouse injured hearts. **a** Heatmap shows the log_10_ (normalised counts +0.01) of selected differentially expressed transcripts (adjusted *p*-value < 0.05). Red—high expression. Yellow—medium expression. Blue—low expression. Selected differentially expressed transcripts are further classified into subcategories of re-innervation (plum), extracellular matrix (ECM) components (yellow), ECM enzymes (turquoise), resolution of inflammation and regeneration (purple), pro-inflammatory mediators (pink), macrophage function (red) and monocyte to macrophage differentiation (green). **b**–**g** Hierarchical clustering reveals dynamic gene signatures. Selected genes from individual clusters are highlighted on the heatmap. **h** Heatmap showing semi-hierarchical clustering of differentially expressed transcripts between regenerative (P1) and scar-forming (P7 and adult, Ad) mouse models at day 7 post-MI. Red—high expression. Yellow—medium expression. Blue—low expression. Selected genes are highlighted. **i**–**k** Unbiased temporal clustering demonstrating dynamically regulated gene sets in macrophages across the phases of scar-based wound healing in the adult mouse heart post-MI. Significant profiles from the inflammatory (**i**), transition (**j**) and reparative (**k**) phases of wound repair are shown (*n* = number of genes within set), with selected genes highlighted. **l**–**n** Unbiased temporal clustering demonstrating dynamically regulated gene sets in macrophages across the time period of regenerative healing in the P1 neonatal mouse heart post-MI. Significant profiles of gene sets upregulated (**l**, **m**) or transiently upregulated (**n**) in regeneration are shown (*n* = number of genes within set). Selected genes are highlighted.

For the fish studies, we used in vivo biotinylation^19^ to specifically biotag nuclei of zebrafish macrophages^20^ derived from injured hearts, thereby enabling us to obtain active (nascent) transcriptomes and to interrogate dynamic gene regulatory landscapes (Supplementary Fig. 3a). Confocal imaging of *TgBAC(mpeg1:BirA-Citrine)*^*ox122*^ adult heart sections revealed GFP staining in macrophages (Supplementary Fig. 3b; fuschia, white arrowheads). Biotagged nuclei were isolated from *TgBAC(mpeg1:BirA-Citrine;βactin:Avi-Cerulean-Rangap)*^*ox133*^ hearts by nuclear pulldown^19^ at 1 dpi, 5 dpi and 14 dpi (cryoinjury) and 1 dpi and 5 dpi (ventricular resection). Scatterplots of normalised read counts with high Pearson’s correlations coefficients demonstrated a high level of reproducibility across replicate experiments (Supplementary Fig. 3c–g and Supplementary Table 1). Differential expression analysis identified 578 downregulated and 3664 upregulated genes following ventricular resection at 1 dpi; 704 downregulated genes and 722 upregulated genes following cryoinjury at 1dpi; 1303 downregulated and 2308 upregulated genes following ventricular resection at 5 dpi, 348 downregulated and 693 upregulated genes following cryoinjury at 5 dpi and 58 downregulated genes and 320 upregulated genes following cryoinjury at 14dpi (Supplementary Fig. 4 and Supplementary Data File 1). Further analysis identified factors involved in specific biological processes including re-innervation (plum-labelled), ECM components (yellow-labelled), ECM enzymes (turquoise-labelled), resolution of inflammation and regeneration (purple-labelled), pro-inflammatory mediators (pink-labelled), macrophage function (red-labelled) and monocyte to macrophage differentiation (green-labelled; Fig. 1a). Macrophages following resection exhibited both acute inflammatory and regenerative responses at 1dpi (Fig. 1a), whereas following cryoinjury macrophages revealed a coordinated response: genes associated with pro-inflammation upregulated at 1 dpi were followed by pro-regenerative/pro-resolution gene signatures at 5 dpi and 14 dpi (Fig. 1a). Using unbiased hierarchical clustering, we identified six cohesive groups of co-expressed factors across different time points and in the different injury settings (Fig. 1b–g): cluster 1 containing genes involved in the initial pro-inflammatory response (*nos2a*, *tril*, *il11b*, *il16*, *il1*β) (Fig. 1b); cluster 2 harbouring ECM remodelling enzymes and MMPs (*lox2lb*, *adam8a*, *mmp14*) as well as molecules associated with resolution of inflammation, growth and proliferation (*il10r*, *tgfβ2*, *ctgfb*, *nrg2b*, *hbegf*; Fig. 1c); cluster 3 implicated in re-innervation, growth and proliferation (*myrf1*, *myelin protein zero P0*, *pdgfr1*, *fgf7* and *fgf14*; Fig. 1d); cluster 4 including, similar to cluster 1, ECM remodelling enzymes and matrix metalloproteases (*mmp23b*, *mmp25*, *mmp15*, *adamts15b*) albeit with persistent expression earlier and across all stages, suggesting a more prolonged mechanism of action (Fig. 1e); cluster 5 comprising pro-inflammatory *il12b*, as well as several cytokine receptors (*marco*, *il17rc*, *nrg1*, *il6r*) previously shown to be necessary for heart regeneration^21,22^ (Fig. 1f) and cluster 6 containing genes predicted to be involved in repair and regeneration, (*tgfβr3*, *bmp10*, *elna*) as well as ECM remodelling (*loxl2a*, *adamts5*, *serpinh1b*; Fig. 1g). These data suggest that a robust immune response is compatible with heart regeneration in zebrafish and the differential responses of macrophages, following resection or cryoinjury insults, may influence downstream wound healing.

In mouse, we carried out bulk RNA-seq of macrophages isolated from P1, P7 and adult hearts by FACS at baseline and days 1, 4 and 7 post-MI (Supplementary Fig. 2 and Supplementary Data File 2). Principal component analyses (PCA) separated P1, P7 and adult macrophage datasets at baseline and post-injury, highlighting divergent transcriptional responses to MI (Supplementary Fig. 5a). Differential expression analysis, between baseline and day 4 post-MI (Supplementary Fig. 5b) and between day 4 and day 7 (Supplementary Fig. 5c), revealed 1500 differentially expressed genes in the adult as compared to 398 and 470 genes in the P1 and P7 models, respectively. Of the genes upregulated in the adult at day 4 post-injury, only 39 were conserved across P1 and P7 samples at the same time-point and analysis of day 4 and day 7 post-injury revealed that while 437, 419 and 217 genes were differentially expressed in the adult, P1 and P7 model respectively, only 43 genes were shared across all samples (Supplementary Fig. 5b, c). These data suggest the macrophage response is less complex with distinct gene-regulatory programmes in neonates compared to the adult. Semi-hierarchical clustering, comparing the regenerative P1 macrophage response to that of the scar-forming P7/adult response at post-injury day 4 (Supplementary Fig. 5d) and day 7 (Fig. 1h), revealed upregulation of canonical mediators of fibrosis, including *Ctgf*, *Tgfβ3*, and *Serpine1*^23^. Other upregulated genes included *Lox* and *Loxl2*, which are associated with cross-linking and stabilisation of collagen fibrils in scar deposition^24^. In contrast, P1 macrophages uniquely upregulated a subset of genes which may be linked to heart regeneration, based on known functions in other tissue-injury settings, including *Il22*^25,26^, *Igfbp4*^27^ and *Ltb*^28^. Statistical over-representation revealed that P7/adult genes at day 7 were associated with gene ontology (GO) terms related to ECM organisation and response to injury and stress (Supplementary Fig. 5e–g). In contrast, P1 upregulated genes were associated with leucocyte activation and functioning of the immune system (Supplementary Fig. 5h). To further understand the differences in the functional responses of macrophages in the P1 and adult hearts, we performed unbiased clustering of adult heart RNA-Seq datasets. Based on their temporal dynamics of gene expression, we identified distinct gene clusters at different phases of myocardial injury and repair (Fig. 1i–k, Supplementary Fig. 5i, j): at day 1 a cluster of 1086 genes associated with production of pro-inflammatory pathways and cytokines (Fig. 1i, e.g. *Il1b, Il6, Nos2*); at day 4 a cluster of 822 genes related to activation of pathways involved in clearance of necrotic debris and antigen presentation (Fig. 1j, e.g. *Apoe, Trem2*) and at day 7 a cluster of 1264 genes, comprised soluble mediators *and* growth factors, which underlie deposition of ECM and tissue remodelling (Fig. 1k, e.g. *Mmp16*; examples from each cluster are shown in Fig. 1i–k, Supplementary Fig. 5i, j). Further clustering of P1 RNA-seq datasets (day 4-7) identified multiple soluble factors with reported mitogenic, pro-survival and angiogenic functions, including *Wnt5a*, previously implicated as a key mediator of intestinal regeneration^29^, and *Bmp7*, involved in neonatal digit regeneration after amputation^30^ (Fig. 1l–n; Supplementary Fig. 5k–m). Next, we analysed genes that were differentially expressed between P1 and P7 stages (Supplementary Fig. 5n and Supplementary Data File 3). Genes upregulated at P7 were primarily associated with ECM organization (Supplementary Fig. 5o), while genes differentially upregulated at P1 were associated with nucleosome assembly and positive regulation of IFN-gamma production (Supplementary Fig. 5p). These results suggest that distinct transcriptional programmes may underlie macrophage function during neonate versus adult mouse cardiac injury and contribute to the transition from regenerative to scar-based repair.

### Macrophages express collagen and collagen-associated genes as potential determinants of scar formation

**Fig. 2:**
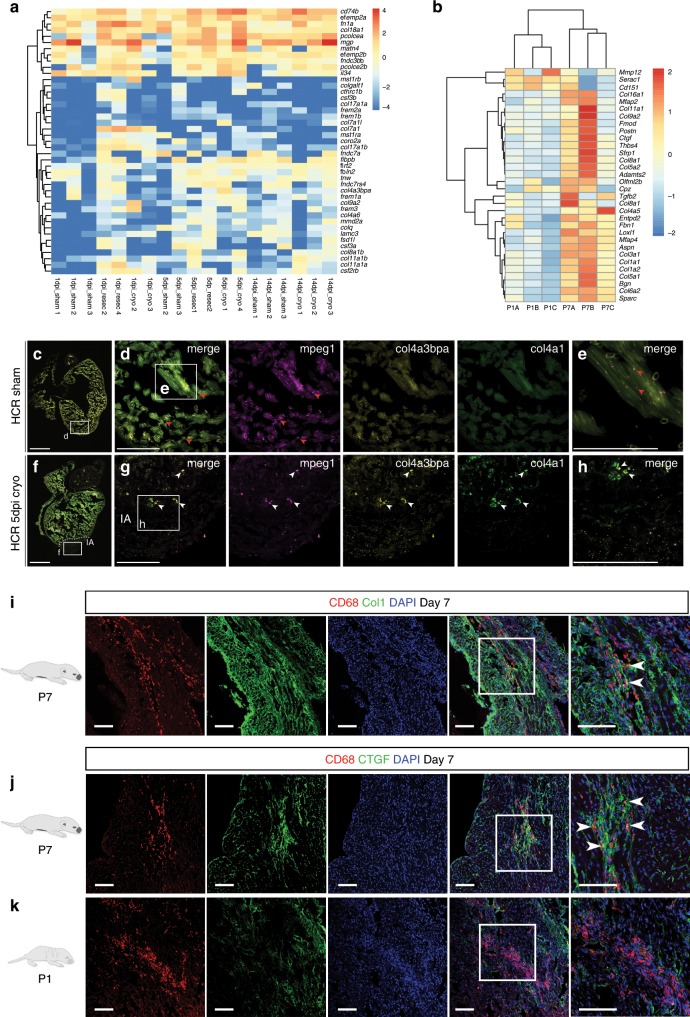
ECM proteins are expressed by macrophages during heart regeneration and scar formation. **a** Heatmap shows the log_10_ (normalised counts +0.01) of differentially expressed transcripts for extracellular matrix (ECM) components. Red—high expression. Yellow—medium expression. Blue—low expression. **b** Heatmap showing expression of ECM components in the regenerating P1 and scar-forming P7 mouse at day 7. ECM genes for comparison were identified from those expressed by adult mouse macrophages during the reparative phase of post-MI (temporal profile Fig. 1k) which also linked to the Proteinaceous Extracellular Matrix GO term. Red—high expression. Yellow—medium expression. Blue—low expression. **c**–**h** HCR confocal imaging of simultaneous *mpeg1* (fuchsia), *col4a3bpa* (yellow) and *col4a1* (green) mRNAs patterns in the heart. Higher abundance of *col4a3bpa* and *col4a1* transcripts in macrophages (white arrowheads) present in the 5 days post cryoinjury heart (**f**, injured area shown by dotted line, white boxes enlarged in **g**, **h** for detail), when compared to sham-operated hearts (**c**, white boxes enlarged in **d**, **e** for detail). Red arrowheads point to macrophages in the sham-operated heart. Scale bar: 200 μm (insets showing high-magnification images, scale bar: 100 μm). dpi: days post injury. IA: injured area. Representative images of *n* = 3 per group. **i** Confocal imaging of immunofluorescence-stained heart cryosections at day 7 post-MI from P7 mice, showing CD68^+^ macrophages (red) in close colocalisation with collagen I (Col1, green) fibrils in the process of scar formation (representative images of *n* = 3, scale bar: 100 μm). **j**, **k** Confocal imaging of immunofluorescence-stained heart cryosections at day 7 post-MI from P1 and P7 mice, showing CD68^+^ macrophages (red) and Connective Tissue Growth Factor (CTGF; green), a soluble pro-fibrotic mediator driving activation of myofibroblasts. As suggested by transcriptional profiling of macrophages in regeneration and scar formation (Fig. 1h), CTGF is expressed widely by macrophages in the infarct zone of the P7 scar-forming heart (**j**), but minimally by macrophages in the P1 site of injury (**k**) (Representative images of *n* = 3 per group, scale bar: 100 μm).

During our transcriptional analyses we observed dynamic, differential expression of collagens and related ECM genes in macrophages from injured zebrafish hearts (Fig. 2a) and between neonatal P1 and P7 stages in mouse, (day 7 post-MI; Fig. 2b), suggesting a direct pro-fibrotic function.

To investigate this further in adult zebrafish, we focused on 5 dpi in the cryoinjury model, at which point there was a significant relative expression of collagen isoforms and associated binding proteins (Supplementary Fig. 6a). It is not known which collagen isoforms predominate during scarring in zebrafish, but we observed that the genes coding for collagen 4a binding protein (*col4a3bpa*) as well as *col7a1l* were upregulated upon injury (Supplementary Fig. 6a, b). The *col4a3bpa* gene encodes three different polypeptides including the canonical Goodpasture antigen binding protein (GPBP), a Ser/Thr kinase. GPBP phosphorylates the non-collagenous domain 1 of type IV collagen and serves to direct collagen molecular and supra-molecular organisation^31,32^. Multiplexed fluorescent in situ hybridisation chain reaction (HCR)^33^ confirmed combinatorial gene expression patterns for *mpeg1*, *col4a3bpa* and its cognate collagen^34^, *col4a1*, in the same heart section at 5dpi, corroborating our transcriptomics data (Fig. 2c–h, white arrowheads).

In the neonatal mouse heart, the mechanisms by which fibrosis is regulated have not been previously studied. A number of genes encoding matrix proteins were expressed by macrophages in the adult heart during the wound healing phase (cluster shown in Fig. 1k). To investigate this further, we compared the expression of these genes within macrophages isolated from P1 and P7 infarcted hearts at day 7 (Fig. 2b). Scar-formation at P7 was associated with upregulation of multiple ECM genes including *Col8a1*, *Col5a2*, *Col6a2*, *Fbn1, Postn*, and *Bgn*, and major fibrillar collagen mRNA isoforms (*Col1a1, Col1a2, Col3a1*; Supplementary Fig. 6c) consistent with a global upregulation of the same collagen isoforms in whole heart samples (Supplementary Fig. 6d). The upregulation of collagens at P7 was accompanied by evidence of collagen-1^+^ fibrils in the infarct region, in contrast to the P1 hearts in which collagen remained sporadic and disorganised (Supplementary Fig. 6e).

In injured P7 hearts CD68^+^ macrophages were frequently observed in close proximity to deposited collagen (Fig. 2i), and were co-localised in the infarct zone with Connective tissue growth factor (CTGF), a matricellular protein with known roles in the activation of myofibroblasts^35^ (Fig. 2j). In contrast, almost no CTGF expression was observed at the site of injury in P1 hearts, despite the presence of macrophages (Fig. 2k). To further investigate collagen expression in macrophages during scarring, we crossed Col1a2CreERT2 mice, which express *Cre* recombinase under the Col1a2 promoter, with R26R-YFP reporter mice. Offspring underwent surgery at P7 and Cre was activated by tamoxifen at day 5 post-MI. At day 7, we detected a small subset of CD68^+^ macrophages that were YFP^+^ within the forming scar (Supplementary Fig. 6f). Together, these data suggest that CD68^+^ macrophages contribute to P7 scar formation by both cell-autonomous and non-cell autonomous production of ECM proteins as a key determinant of scarring.

### Macrophages directly contribute to collagen deposition and scar formation

**Fig. 3:**
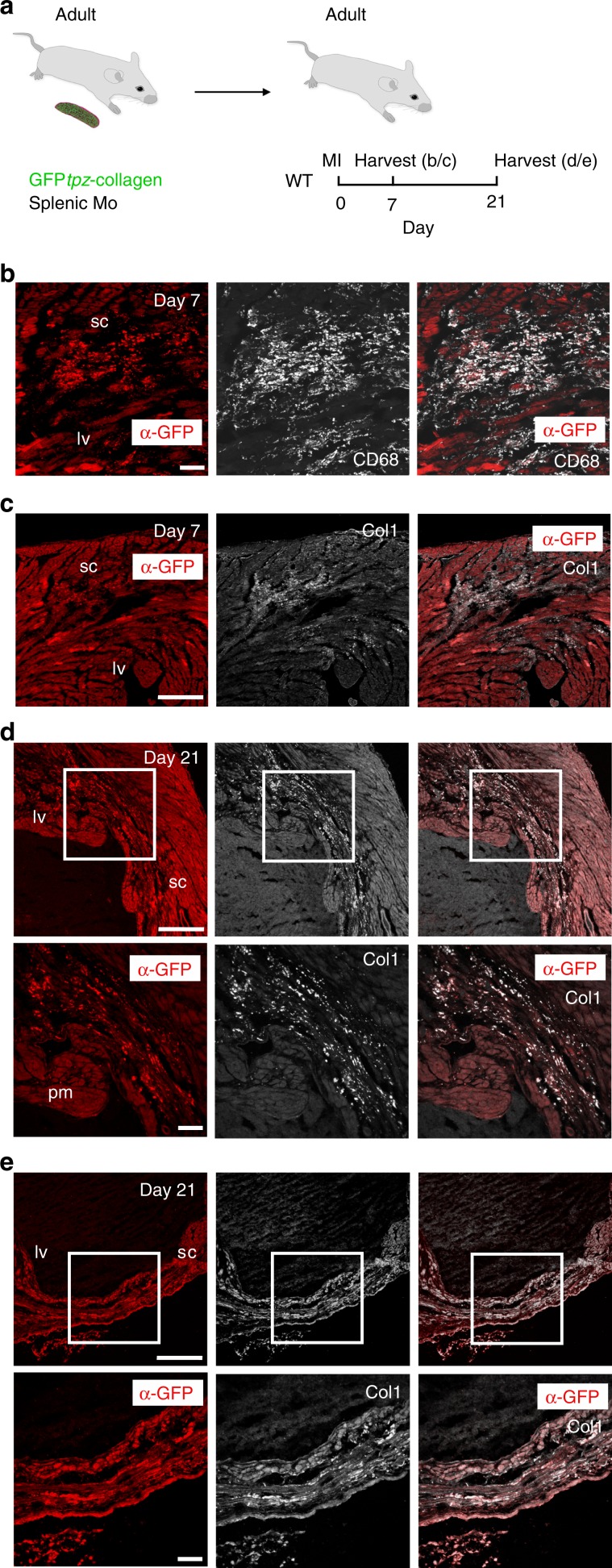
Adult mouse splenic GFP^+^ monocytes contribute collagen to the scar post-MI. **a** Experimental design for adoptive transfer: monocytes were isolated by magnetic column purification from the spleens of adult GFP*tpz*-collagen^+^ mouse donors. At the time of MI surgery, 50 ± 10 × 10^3^ monocytes/PBS, or PBS alone, were transferred to wild type (WT) recipients by intracardiac injection. Adult recipient mice were then harvested at either day 7 (**b**, **c**) or day 21 (**d**, **e**) for immunostaining. **b** At day 7 post-MI, combined immunostaining for α-GFP (red; to exclude autofluorescence from monitoring GFP-alone) and α-CD68 revealed GFP^+^ macrophages within the scar region (sc) of the left ventricle (lv). **c** Co-staining for collagen-1 (white, Col1) revealed GFP^+^ collagen deposits within the same scar region. **d** By day 21, there was increased GFP^+^ collagen deposition within the scar (white inset boxes in upper panels shown at higher magnification in corresponding panels below). **e** GFP^+^ collagen fibres were evident within regions of transmural scar, as detected by combined α-GFP and Col1 staining (white inset boxes in upper panels shown at higher magnification in corresponding panels below). lv, left ventricle, pm, papillary muscle; sc, scar. Scale bars: **b**, **c** upper panel, **d** upper panel 100 μm; **c** lower panel, **d** lower panel 50 μm. Representative images of *n* = 3 per group.

To address whether macrophages contribute directly to collagen deposition in the P7 and adult mouse heart post-MI, we utilised a transgenic mouse line that expresses a collagen-GFP fusion protein to fluorescently label type-1 collagen fibrils^36^. We initially characterised GFP*tpz*-collagen deposition in adult mice post-MI (Supplementary Fig. 7a–e) and observed GFP^+^ signal within the infarct region (Supplementary Fig. 7a) and GFP-labelled fibres positive for collagen-1 within the scar (Supplementary Fig. 7b–e). To assess whether GFP*tpz*-collagen-labelled macrophages may contribute directly to the forming scar we carried out an adoptive transfer experiment by injecting adult GFP*tpz*-collagen positive donor monocytes, isolated and purified from the spleen (50 × 10^3^ GFP^ +^ cells), into the peri-infarct myocardium of adult wild type littermates undergoing MI surgery (Fig. 3a). At day 7 post-MI, GFP^+^ signal was observed within the scar region which colocalised with CD68^+^ macrophages (Fig. 3b) and collagen-1 antibody staining (α-Col1; Fig. 3c). By day 21, the incidence of GFP^+^/collagen-1^+^ signal had increased and was detected within multiple regions of the transmural scar including fibrillar structures (Fig. 3d, e), thus providing strong evidence of a contribution of macrophage-derived collagen to the scar post-MI. We further tested the ability of macrophages to deposit collagen in vitro and established co-cultures of isolated bone-marrow derived monocytes from GFP*tpz*-collagen mice with L929 fibroblasts that were activated by a cytokine cocktail of TNF-α, Il-1β, IL4 and TGFβ. This revealed GFP*t*pz^+^ fibres associated with CD68^+^  macrophages (Supplementary Fig. 7f–h), which colocalised with α-GFP (Supplementary Fig. 7i–k) and α-Col1 immunostaining (Supplementary Fig. 7l–n) and correlated with the number of macrophages within the culture (Supplementary Fig. 7o–q), providing further evidence of macrophage-derived collagen deposition  under activated (inflammatory/reparative) conditions.

To assess the relative proportions of macrophage-derived collagen versus a myofibroblast source, we carried out flow cytometry of GFP^+^ cells isolated from the hearts of Col1a1-GFP mice^37^ at day 7 post-MI. Macrophages and fibroblasts were identified within the GFP^+^ population as F4/80^+^ and anti-Feeder Cells-APC^+^, respectively. Macrophages comprised 5.54% and fibroblasts 75.34% of the total GFP^+^ cell population (Supplementary Table 2 and Supplementary Fig. 8a). Interestingly, whilst macrophages made up only 0.45% of the total cells in the heart compared to fibroblasts which accounted for 6.1%, of the total macrophage population, 46.2% were GFP^+^ compared to 22.5% of fibroblasts (Supplementary Table 2). These data suggest that while fibroblasts are likely to account for the majority of collagen-deposition in terms of the abundance of Col1a^+^ cells, the macrophage contribution is not insignificant, amounting to around 6.8% of the total.

**Fig. 4:**
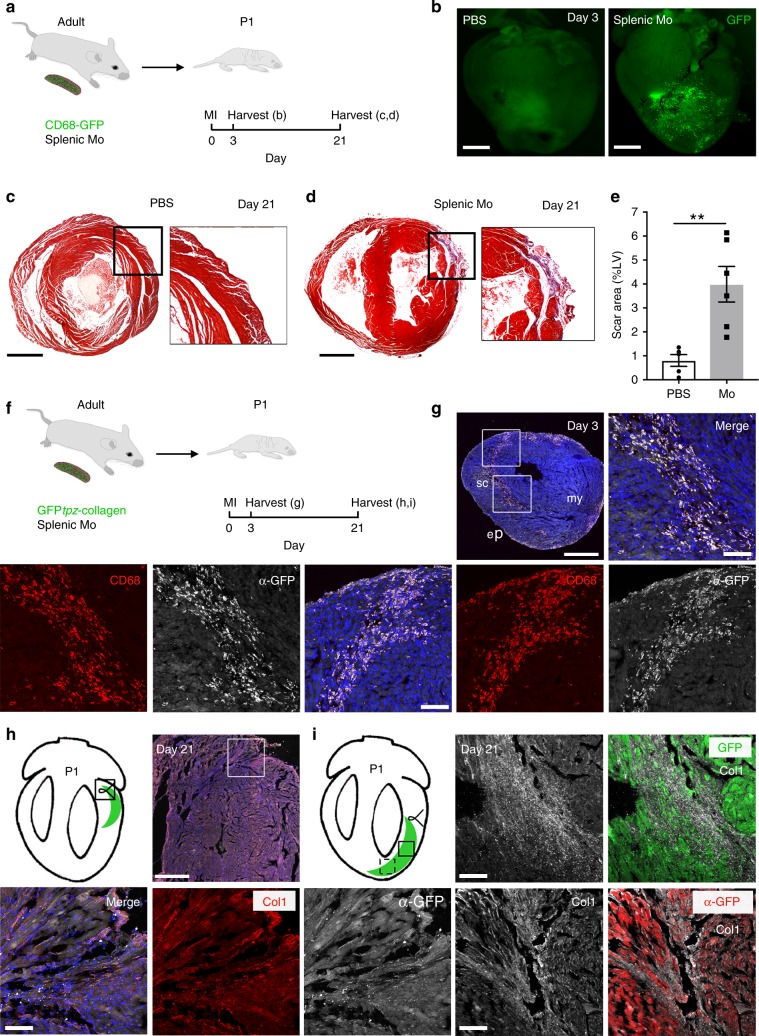
Adult monocytes disrupt regeneration of P1 mouse heart and deposit collagen within a post-MI scar. **a** Adult splenic monocytes from hCD68-GFP^+^ donors were transferred by intracardiac injection at time of MI surgery (50 ± 10 × 10^3^ cells). P1 neonates were then harvested at day 3 (for imaging) or day 21 (for histology). **b** GFP Fluorescence imaging demonstrating adoptively transferred GFP^+^ monocytes were present in the anterior wall of the recipient P1 at day 3 post-MI (scale bar: 1 mm). **c** Masson’s trichrome staining of heart sections showing that at day 21 post-MI, P1 neonates which received PBS vehicle showed histological evidence of regeneration. **d** P1 neonates which received adult monocytes show residual scar formation. Scale bar: 1 mm. **e** Adoptive transfer of adult monocytes led to a significant increase in scar size at 21 days, measured as percentage of the left ventricular area (scar percentage 3.99 ± 0.74% vs 0.81 ± 0.25%, *n* = 5 in PBS group, *n* = 6 in monocyte group, **p *<* 0.01, data are mean ± SEM, 2-tailed, unpaired Student’s *t*-test). **f** Splenic monocytes transferred from adult GFP*tpz*-collagen mice to P1 recipients undergoing MI. **g** Confocal imaging of immunofluorescence-stained heart cryosections at day 3 post-MI revealed emergent GFP^+^ fibres within the scar region and a subset of α-CD68^+^ macrophages co-labelled for GFP indicating that they had arisen from donor monocytes and had activated the GFP*tpz*-collagen 1. Scale bars, 500 μm (low power panel), 100 μm (high power panels). **h**, **i** By day 21 post-MI, recipient P1 mice revealed GFP^+^ scars within the non-regenerated myocardium (schematised hearts; black inset boxes relate to immuno-stained regions in the vicinity of the ligating suture) (**h**) or within the injury area (top two panels in **i**; black dashed inset box relates to injury region depicted in bottom two panels in **i**). Both GFP fluorescence and α-GFP staining revealed evidence of donor-monocyte-derived GFP^+^ collagen fibrils which were Col1^+^ within the scar at day 21 post-MI. Scale bars, 500 μm (low power panel), 100 μm (high power panels). ep, epicardium; my, myocardium; P1, post-natal day 1; sc, scar. Representative images of *n* = 3 per group. Source data are provided as a Source Data file.

We next tested whether adoptive transfer of CD68-GFP^+^ labelled adult splenic monocytes into injured P1 hearts could evoke fibrosis and collagen deposition in a regenerative environment (Fig. 4a). At day 3 post-MI, donor GFP^+^ monocytes/macrophages were visualised in the infarct zone of the P1 host (Fig. 4b). At 21 days post-MI, P1 recipient hearts containing adoptively-transferred monocytes had significantly larger areas of scar deposition than controls (Fig. 4c–e; scar percentage 3.989 ± 0.743% vs 0.807 ± 0.246%, *n* = 5 in PBS group, *n* = 6 in monocyte group, **p *=* 0.0046, data are mean ± SEM, 2-tailed, unpaired Student’s *t*-test), suggesting monocyte-derived CD68^+^ macrophages can drive the processes of irreversible scarring and pro-fibrotic healing. Next, we adoptively transferred adult splenic monocytes derived from GFP*tpz*-collagen mice into wild type P1 recipients at the time of MI (Fig. 4f). We initially confirmed detection of CD68^+^/GFP^+^ macrophages in the infarct zone of the P1 recipient heart at day 3, embedded within the collagen-1^+^ scar region (Fig. 4g). At day 21 post-MI we observed GFP^+^/Col1^+^ fibres within both the proximal and more remote regions of injury (Fig. 4h, i).

These data suggest that adult mouse monocytes, which give rise to differentiated macrophages post-MI, can contribute collagen to the scar and that cell-autonomous production of collagen is a component of the pro-fibrotic monocyte/macrophage response.

**Fig. 5:**
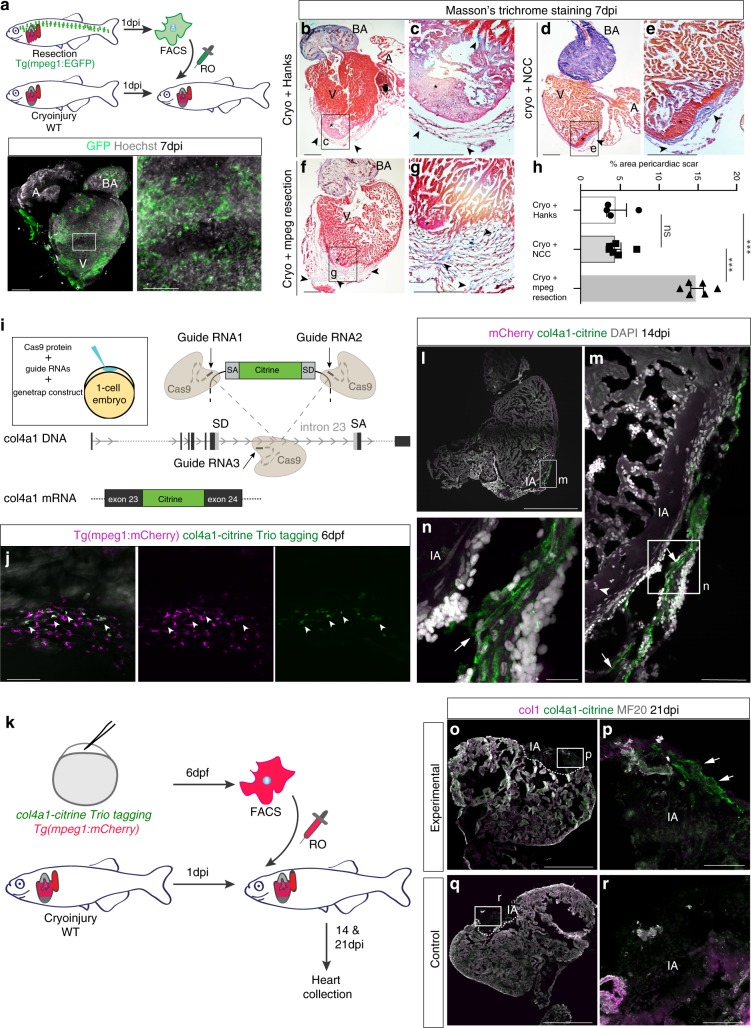
Zebrafish macrophages directly contribute to transient scar formation by collagen synthesis. **a** Adoptive transfer of GFP^+^ macrophages derived from a resection heart into a cryoinjured heart via retro-orbital (RO) injection. Wholemount heart showing transplanted macrophages in 7 days post injury (dpi) recipient hearts, hoechst-stained nuclei in grey. Scale bars: 200μm, high-magnification 100 μm. **b**–**g** Masson’s trichrome staining of Hanks-injected (**b**; high-magnification in **c**), neural crest cells (NCC)-transplanted (**d**; high-magnification in **e**), and macrophages-transplanted (**f**; high-magnification in **g**) hearts showing pericardiac fibrosis (blue, arrowheads) around the cryoinjured area (pink, black asterisk). Scale bars: 200 μm, high-magnification: 50 μm. **h** Quantification of scar fibrosis (percentage of total section area, 6 sections per heart: hanks-injected 4.611 ± 1.26%, *n* = 4 vs macrophage-transplanted 14.87 ± 0.81%, *n* = 6; ***p *<* 0.0001; NCC-transplanted 4.53 ± 0.643%, *n* = 6 vs macrophage-transplanted 14.87 ± 0.81%, *n* = 6; ***p *<* 0.0001; hanks-injected 4.611 ± 1.26%, *n* = 4 vs NCC-transplanted 4.53 ± 0.643%, *n* = 6; *p* *=* 0.951, ns, not significant). 2-tailed, unpaired Student’s *t*-test, data are mean ± SEM. **i** “Trio” tagging approach: Cas9 protein, guide RNAs and genetrap construct coding for Citrine, are injected in 1-cell stage embryos. Double-strand breaks are generated in the *col4a1* intron and the donor construct, resulting in integration of *Citrine* ORF flanked by splice acceptor (SA) and donor (SD) sites, ultimately ensuing a col4a1-Citrine fusion protein. **j** Mosaic expression of col4a1-Citrine observed in 6 dpf macrophages (arrowheads) of “trio” tagging-injected embryos. Scale bar: 50 μm. **k** Adoptive transfer of mCherry^+^ macrophages, FAC-sorted from “trio” tagging-injected embryos, transplanted into WT cryoinjured heart via RO injection. **l**–**n** 14 dpi WT hearts transplanted with col4a1-Citrine mCherry macrophages. Very few transplanted macrophages (**m**, arrowhead) are still present in the injury region (dashed line and IA in **l**, IA in **m**, **n**). Macrophage-deposited mosaic “green” scar is observed extracellularly, near the injured area (arrows **m**, **n**, DAPI-stained nuclei in grey). (**o**–**r**) 21 dpi WT transplanted hearts reveal a mosaic scar (arrows), also stained for collagen 1 (fuschia), peripheral to the MF20-negative injury area (dashed line and IA in **o**, IA in **p**), not observable in control conditions (**q**, **r**). Scale bars **l**–**n**: 200 μm, high-magnification: 100 μm). Representative images of *n* = 3 per group. Source data are provided as a Source Data file.

To further investigate macrophages in different injury environments, we performed analogous adoptive cell transfer experiments in adult zebrafish, by transplanting macrophages obtained from the resection model into the post cryoinjured (scar-inducing) heart. We performed ventricular resection injury in *Tg(mpeg1:EGFP)*^*gl22*^ transgenic zebrafish which report macrophages via GFP^+^ fluorescence^38^. GFP^+^ macrophages were isolated 1 day post resection from adult hearts by FACS (Supplementary Fig. 8b), and transferred into a cryoinjured wild type (WT) zebrafish heart, at 1 dpi by retro-orbital injection^39^ (Fig. 5a). Immunostaining of the transplanted heart 6 days after cell transfer (7 dpi) confirmed the presence of GFP^+^ cells throughout the injured heart (Fig. 5a). Masson’s trichrome and AFOG staining for collagen highlighted the fibrotic response around the injury site in 7 dpi cryoinjured hearts injected with (1) Hanks buffer-only (Fig. 5b, c and Supplementary Fig. 9a), (2) purified neural crest cells (NCCs) derived from *Gt(foxd3-Citrine)*^*ct110*^ 16-somite stage embryos^40^ as controls (Fig. 5d, e and Supplementary Fig. 9b) and (3) mpeg1^+^ macrophages (Fig. 5f, g and Supplementary Fig. 9c). Cryoinjured hearts transplanted with macrophages revealed a significant increase in collagen staining located around the periphery of the wound relative to the Hanks buffer and NCC-injected controls (Fig. 5b–h and Supplementary Fig. 9a–d; Hanks control hearts: 4.611 ± 1.26%, *n* = 4 vs macrophage transplanted hearts: 14.87 ± 0.81%, *n* = 6; ***p *<* 0.0001, 2-tailed, unpaired Student’s *t*-test; neural crest cells transplanted control hearts: 4.53 ± 0.643%, *n* = 6 vs macrophage transplanted hearts: 14.87 ± 0.81%, *n* = 6; ***p *<* 0.0001, 2-tailed, unpaired Student’s *t*-test).

To test whether macrophages can directly contribute collagen to the forming scar in zebrafish, we performed further adoptive cell transfer experiments using macrophages expressing fluorescent Citrine-tagged endogenous *col4a1* generated by CRISPR/Cas9-mediated intron-based protein trapping (*Tg(mpeg1:mCherry)*^*gl23*^ ^38^; *col4a1-Citrine* “trio” tagging; Fig. 5i–r and Supplementary Fig. 9e). At 6 days post fertilization (dpf), mosaic expression of Citrine-tagged *col4a1* was detected intracellularly in macrophages (Fig. 5j). These macrophages were FACS-isolated (Supplementary Fig. 8b) and transferred into a 1 dpi cryoinjured wild type zebrafish heart. Transplanted hearts were collected at 14 and 21 dpi (Fig. 5k–r and Supplementary Fig. 9e). Immunostaining revealed that the extracellular fibrotic response around the injury area of the cryoinjured heart was positive for col4a1-Citrine and was fibrillar in nature at 14 dpi (Fig. 5l–n and Supplementary Fig. 9e) and 21 dpi (Fig. 5o, p). The majority of tagged col4a1 was deposited proximal to the injury, within the overlying epicardial region, as opposed to directly within the major site of fibrosis, suggesting a possible distinction between macrophage-collagen deposition and that predominantly laid-down by activated myofibroblasts. In contrast, Citrine expression was not observed in hearts transplanted with non-tagged macrophages where intronic sgRNA was omitted in the injection cocktail (Fig. 5q, r). Thus, these data reveal that macrophages not only express collagens at the intracellular level but can also contribute to the forming extracellular scar by direct collagen deposition.

**Fig. 6:**
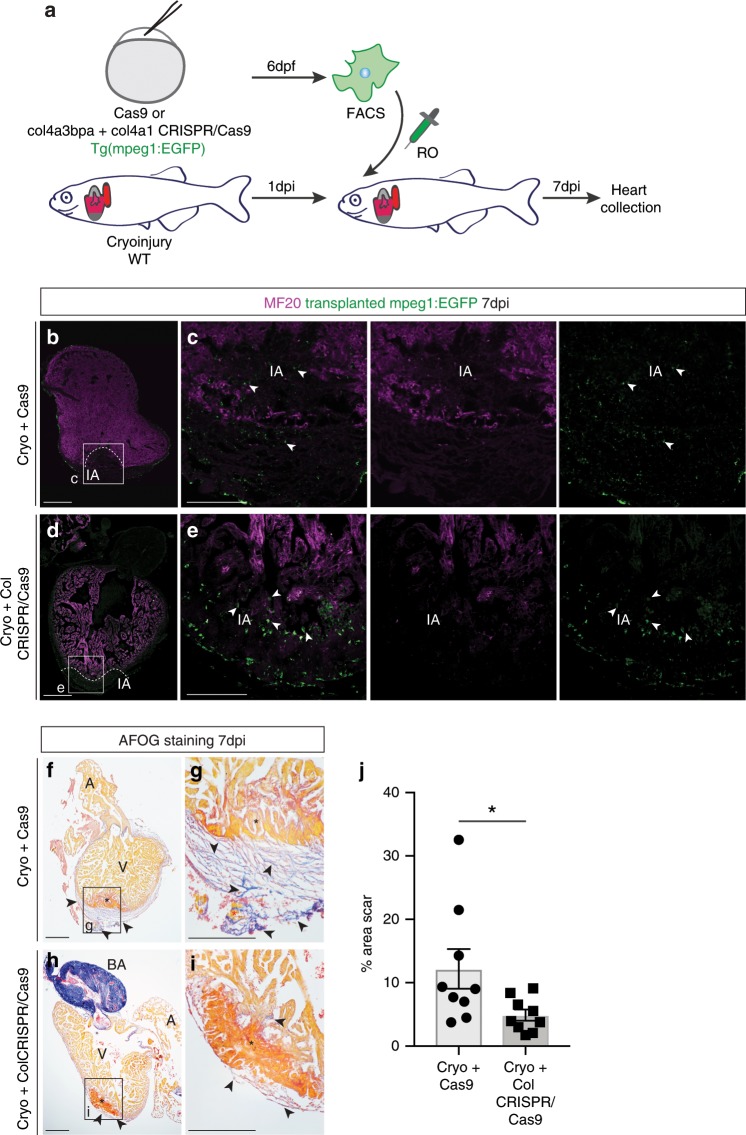
Targeting *col4a1* and *col4a3bpa* in macrophages reduced scarring in zebrafish cryoinjured hearts. **a** Schematics of adoptive transfer of GFP^+^ macrophages sorted from embryos injected with Cas9 only or *col4a3bpa* plus *col4a1* sgRNAs/Cas9 and transplanted into a cryoinjured heart via retro-orbital (RO) injection. **b**–**e** Confocal imaging of anti-MF20 (fuschia) and anti-GFP (green) antibody-stained WT recipient hearts of Cas9 only (**b**, white boxes enlarged in **c** for detail) and *col4a3bpa* plus *col4a1* sgRNAs/Cas9 (**d**, white boxes enlarged in **e** for high-magnification) transplanted macrophages (GFP^+^) collected 7 days post injury (dpi). Arrowheads point to GFP^+^ transplanted macrophages that are located in the site of injury (IA). Scale bar: 200 μm (insets showing high-magnification images, scale bar: 100 μm). Dotted line demarcates the MF20-negative injured area. Representative images of *n* = 3 per group. (**f**–**i**) AFOG staining of representative images showing healthy myocardium (yellow), injured myocardium (orange) and collagen (blue). Excess scar tissue (arrowheads) at the periphery of the cryoinjured area (black asterisk) is seen in hearts transplanted with 6 days post fertilization (dpf) GFP^+^ macrophages injected with Cas9 only (**f**, with high-magnification inset in **g**). Hearts transplanted with *col4a1*+co*l4a3bpa* CRISPR/Cas9 macrophages show reduced collagen staining (**h**, with high-magnification inset in **i**); red asterisk, myocardial dead tissue removed from the injury region. Representative images of *n* = 3 per group. **j** Quantification as a percentage of total section area (6 sections per heart) show significantly less scar fibrosis in the group of cryoinjured hearts transplanted with *col4a1*+*col4a3bpa* CRISPR/Cas9 macrophages (Cryo+Cas9: 12.18 ± 3.12%, *n* = 9 vs Cryo+Col CRISPR/Cas9: 4.91 ± 0.88%, *n* = 9; *p *=* 0.039; data are mean ± SEM, 2-tailed, unpaired Student’s *t*-test). Scale bar: 200 μm; high-magnification insets scale bar: 50 μm; A, atrium; V, ventricle; BA, Bulbus Arteriosus. Source data are provided as a Source Data file.

To determine whether macrophage-derived collagen is functionally relevant for scar formation in zebrafish, we performed further adoptive cell transfer experiments using macrophages carrying genetic deletions of *col4a3bpa*, upregulated at 5dpi in the cryoinjury setting, and its cognate collagen, *col4a1* (Fig. 6a). Transgenic *Tg(mpeg1:EGFP)*^*gl22*^ zebrafish embryos were injected with *Cas9* mRNA only (Cas9-only controls) or with Cas9 mRNA combined with gRNAs targeting both *col4a3bpa* and *col4a1* genes (Supplementary Fig. 9f). Efficiency of the gRNAs and editing events were tested using HRMA (Supplementary Fig. 9g) and the penetrance of mutations assessed by profiling CRISPR-mediated somatic indels using targeted NGS (Supplementary Fig. 9h, i). Macrophages were isolated by FACS (Supplementary Fig. 8b) from genome-edited embryos at 6 dpf, by which point the kidney marrow is sufficiently established as the site of adult haematopoiesis^41,42^. QPCR quantification showed a significant reduction in intact transcripts of both *col4a3bp* and *col4a1* in the transiently edited F_0_ macrophages (Supplementary Fig. 9j), which were transferred into a 1dpi cryoinjured wild type zebrafish heart. Antibody staining of transplanted hearts at 7dpi was performed to confirm the presence of GFP^+^ cells in the MF20-negative/dead muscle area of the injured hearts transferred with either Cas9-only (Fig. 6b, c) or *col4a3bpa*/*col4a1* CRISPR/Cas9-targeted macrophages (Fig. 6d, e). Transplantation of *col4a3bpa*/*col4a1*-deficient macrophages revealed a significant decrease in AFOG collagen staining located around the periphery of the wound (CRISPR/Cas9: 4.906 ± 0.887%, *n* = 9; Cas9 only: 12.180 ± 3.122%, *n* = 9; *p *=* 0.039, 2-tailed, unpaired Student’s *t*-test) (Fig. 6f–j and Supplementary Fig. 9k, l), which was equivalent to that observed in controls, injected with Hanks buffer only (Supplementary Fig. 9m, control hearts: 4.611 ± 1.258%, *n* = 4; CRISPR/Cas9: 4.906 ± 0.887%, *n* = 9; n.s. *p* = 0.855, 2-tailed, unpaired Student’s *t*-test). The reduction in scarring, following functional targeting of a macrophage collagen and its associated binding protein, strongly supports the notion that macrophages contribute directly to scar formation via collagen deposition.

## Discussion

Effective cardiac regeneration depends not only on the degree of regenerative competence of the injured heart but also on the modulation of the immediate fibrotic-based repair into a transient response^43^. Adjusting the balance between a pro-fibrotic versus a pro-regenerative environment is key to a successful regenerative outcome. Recent studies have revealed that macrophages are essential players in the regeneration of the salamander limb^12^, adult zebrafish tail fin^16^ and neonatal mouse heart^10^. Studies in zebrafish have also reported impaired heart regeneration upon a decreased macrophage response^11,13,15,44,45^. However, the molecular programmes deployed by macrophages to either dampen adverse effects of inflammation in an environment permissive for the positive effects of the immune response or to directly exacerbate fibrosis during wound healing remain unknown.

Here we profiled the transcriptional state of macrophages responding to distinct injury cues at different stages of zebrafish heart regeneration and between neonatal and adult mouse hearts post-MI. Temporal characterisation of the cellular and molecular immune responses revealed precise timings for the recruitment of myeloid cells and the establishment of a dynamic immune response in regenerative compared with scar-forming environments. In adult zebrafish, the response was sufficiently robust to argue against existing dogma that the capacity to regenerate is incompatible with a significant immune response after injury^10,46–48^. Subsequent transcriptional analyses identified critical differences in the macrophage regulatory profiles when activated in resection versus cryoinjury conditions. In zebrafish, a synchronised, simultaneous pro- and anti-inflammatory response was coincident with the rapid regenerative process occurring after ventricle resection. Conversely, in the cryoinjury setting, an initial pro-inflammatory response was superseded by anti-inflammatory macrophages to ensure effective regeneration and prevent permanent fibrosis. In the mouse, the global macrophage transcriptional response in P1 neonates was significantly dampened relative to an intermediate state at P7 and highly active response in the adult immediately post-MI.

Strikingly, our transcriptional analyses in zebrafish and mice demonstrated that macrophages expressed collagen and collagen-associated gene isoforms in addition to *bona fide* fibrotic signature genes following cardiac injury. Myofibroblasts, defined as periostin-expressing cells, arising from Tcf21-derived tissue-resident fibroblasts^49^, have traditionally been considered the exclusive source of collagen deposition during scar formation^50^. In-turn, macrophages have been shown to enhance the survival and activation of myofibroblasts, via the release of soluble factors such as TGFβ and FGFs^50^, and thus indirectly influence scarring. Our data strongly suggest that macrophages directly contribute collagen to the forming scar. In mice, adoptive transfer of purified GFP*tpz*-collagen labelled splenic monocytes revealed expression of the fusion-protein within the adult and neonatal heart and GFP^+^ collagen-fibre deposition within the forming scar; whilst in zebrafish, collagen 4a-tagging revealed collagen deposition within the periphery of the cryoinjured wound following the adoptive transfer of macrophages. Moreover, transfer of macrophages genetically depleted of collagen 4a3 binding protein (*col4a3bpa*) and its cognate collagen 4a1 (*col4a1*) revealed a statistically significant reduction in pericardiac scar formation post-cryoinjury. These results implicate macrophages in contributing directly to cardiac fibrosis via autonomous collagen deposition. Importantly this is conserved across species (fish and mammals) and challenges current dogma that the post-cardiac injury scar is laid down exclusively by myofibroblasts.

The adoptive cell transfer of macrophages derived from resected zebrafish hearts transplanted into cryoinjured recipient ventricles revealed excessive scar formation and suggests that macrophages exhibit a high degree of plasticity; altering their function when transferred from a more regenerative into a more scar-inducing environment. This contrasted with the situation in mice, whereby transfer of monocytes derived from adult mouse spleen into P1 neonatal recipient hearts was sufficient to induce scar formation, irrespective of the local regenerative environment; suggesting that adult monocyte-derived macrophages are irreversibly committed towards a fibrotic programme. The reciprocal experiment, transferring P1 (pro-regenerative) monocytes/macrophages into adult heart, would clearly be of interest as to whether this might influence the extent of scarring in the adult heart. However, the theoretical number of donor cells that would need to be isolated from P1 mice to impact on (potentially over-ride) the host population of pro-fibrotic adult macrophages makes this technically unfeasible. The apparent plasticity of differentiated macrophages in adult zebrafish is consistent with studies revealing how the epigenetic state of transplanted bone marrow precursors and differentiated macrophages can be reprogrammed when transferred into a new organ^51,52^. Such plasticity allows macrophages to quickly adapt and regulate different and often opposing outcomes such as regeneration versus fibrotic repair. In contrast, the apparent hard-wiring of adult mouse macrophages following cardiac injury may underpin the default pro-fibrotic wound healing in the mammalian heart and contribute to the lack of regenerative capacity.

Our work adds significantly to the understanding of the cellular and molecular basis of scar formation, in the context of cardiac repair and regeneration, and positions macrophages as key players that contribute directly to the fibrotic response following injury. The question remains as to why macrophages may act as collagen depositing cells during cardiac wound healing and fibrosis? We speculate that in the mouse, since the infiltration of macrophages and their migration to the site of injury is a rapid initiating event during the repair phase post-MI, they may serve to ensure the initial patching of the infarct region to offset ventricular rupture. Thereafter, activation of cardiac fibroblasts ensures a further round of myofibroblast-derived collagen deposition to progress and complete mature scar formation. In zebrafish, the spatial localisation of the tagged macrophage-derived collagen around the wound periphery may act to seal the injury and could serve as an ECM scaffold for the regrowth of epicardium, a critical event prior to myocardial regeneration in zebrafish^53,54^. The functional relevance of macrophage-derived collagen production in terms of cardiovascular outcomes remains to be investigated in the future. A comprehensive understanding of the regenerative environment in which the innate immune response persists but is permissible for regeneration, alongside targeting of macrophage-induced pro-fibrotic pathways, is poised to assist in the development of immunomodulation therapies for patients suffering from acute MI.

## Methods

### Animal maintenance and strains

This study was carried out in accordance to procedures authorized by the UK Home Office in accordance with UK law (Animals Scientific Procedures Act 1986) and approved by the Research Ethics Committee of the University of Oxford.

Wild type, *TgBAC(mpeg1:BirA-Citrine)*^*ox122*^ ^20^, *Tg(βactin:Avi-Cerulean-RanGap)*^*ct700a*^ ^19^, *TgBAC(mpeg1:BirA-Citrine;*β*actin:Avi-Cerulean-Rangap)*^*ox133*^ ^20^, *Gt(foxd3-Citrine)*^*ct110*^ ^40^, *Tg(mpeg1:EGFP)*^*gl22*^ ^38^ and *Tg(mpeg1:mCherry)*^*gl23* ^^38^ zebrafish were bred and maintained in accordance with established protocols^55^.

Wild-type CD1 mice (Harlan), Col1a2-CreERT2, Col1a1-GFP, R26R-tdTomato, R26R-YFP, hCD68-GFP and GFP*tpz*-collagen mice were maintained under specific pathogen-free conditions at the University of Oxford, UK. Where indicated, pups from Col1a2-CreERT2 male x R26R-YFP female matings were injected at day 5 post-MI with 50 μl of 10 mg/ml tamoxifen (total dose 0.5 mg) dissolved in peanut oil. Pups were harvested at the time point indicated and genotyped for Cre and YFP.

### Cardiac injury models

Cardiac injuries were carried out in 4–12-month old zebrafish^6–8^. Briefly, cryoinjury was performed by application of a cryoprobe frozen with liquid nitrogen to the surface of the exposed ventricle until the probe was fully thawed, damaging approximately 20% of the ventricle. For ventricle resection, 20% of the apex of the ventricle was surgically removed, resulting in the immediate formation of a blood clot. Exposing the ventricle, without injury, was performed for sham controls.

Permanent ligation of the left anterior descending coronary artery to induce MI in the adult mouse was performed^56^. For neonatal MI, mice were anaesthetized using isoflurane 2% and then cooled on ice to induce cardio-respiratory arrest^57^. A lateral thoracotomy was then performed, and a 7-0 prolene suture (Ethicon) then tied around the left anterior descending coronary artery to induce MI. The chest and skin were closed with 7-0 prolene, and pups were re-warmed under a heat lamp before being returned to the mother.

### Total RNA extraction and Nanostring nCounter gene expression assay

Whole zebrafish hearts, including the atrium, bulbus arteriosus, and ventricle, were collected and stored in RNAlater Stabilization Solution (Thermo Fisher Scientific) at −80 °C. Samples were collected in triplicates. Fresh-frozen hearts were mechanically dissociated in lysis buffer using a handheld pellet pestle (Sigma-Aldrich). RNA extraction was subsequently performed using the Purelink RNA Mini Kit (Thermo Fisher Scientific). DNase treatment was carried out on samples using the TURBO DNA-free Kit (Thermo Fisher Scientific). RNA purity and concentration were assessed using NanoDrop 2000 (Thermo Fisher Scientific). The nCounter gene expression assay (NanoString Technologies, Seattle, WA) was performed as previously described^58^ using 150 ng of total RNA per heart. Gene expression codesets (designed and produced by NanoString Technologies, Seattle, WA), hybridisation buffer and total RNA were hybridised in a thermocycler for 18 h at 65 °C prior to being processed in the nCounter Prep Station. Data collection using the Digital Analyzer was performed using the maximum field of view setting. Graphs were generated using R package. Raw probe-mRNA counts were normalized to β-actin to compensate for differences in heart size. Statistical significance was calculated by 2-tailed, unpaired Student’s *t*-test; *p < 0.05.

### Quantitative real-time PCR

Total RNA was isolated from mouse hearts using the Qiagen RNeasy Mini Kit (Qiagen). Complementary DNA was synthesized using the Reverse Transcription System (Promega), following the manufacturer’s instructions and used for quantitative real-time PCR using SYBR Green on an ABI 7900 for the following genes: *Col1a2, Col1a2, Col1a3*. Fold change was determined by applying the 2^−ΔΔCt^ method. The following primer sequences were used: *Col1a1* FWD-GCAAGAGGCGAGAGAGGTTT, REV-GACCACGGGCACCATCTTTA, *Col1a2* FWD-CTGGAACAAATGGGCTCACTG, REV-CAGGCTCACCAACAAGTCCTC, *Col3a1* FWD-ACGTAGATGAATTGGGATGCAG, REV-GGGTTGGGGCAGTCTAG.

Cas9-only and *col4a3bpa*/*col4a1* CRISPR/Cas9-targeted zebrafish macrophages were FAC-sorted and RNA extraction and cDNA synthesis carried out using the RNAqueous^®^-Micro Kit (Life Technologies) and Superscript III Reverse Transcriptase (Invitrogen), respectively. Quantitative PCR was performed using Fast SYBR Green Master Mix (Applied Biosystems) on a StepOnePlus Real Time PCR system (Thermo Fisher Scientific). Gene-specific primers used for *col4a1* were FWD1-CAAAGGAACTGATGGGCAAC; REV1-AGCTCCTTTTGTAACATCACATTC; FWD2-TCAGGTTTTCAAGGAGAGCC; REV2-TTTGGGACCTGGAAAAGACC; FWD3-ACTTCAAGAACACATTTGCGTC; REV3-GATGGAACTGCCTTAGTTAACAC. Gene-specific primers used for *col4a3bpa* were FWD1-GTGGACAAACTACATTCATGGC; REV1-GCCGTACTCCTTCTCATCTG; FWD2-CGGCAAACTCAGTAAGTGGAC; REV2-TGAGAATATTGCCCTTCAGCG; FWD3-AGAAGGAGTACGGCTGTAGAG; REV3-AGATGCTGTCGTTCACACTG. Expression levels were normalised to β-actin and fold change was determined by applying the 2^−ΔΔCt^ method.

### Zebrafish biotagged nuclei isolation

Biotagged nuclei were isolated as previously described^19^. Briefly, *TgBAC(mpeg1:BirA-Citrine;*β*actin:Avi-Cerulean-Rangap)*^*ox133*^ operated adult hearts (*n* = 1 per sample) were washed and incubated on ice in hypotonic buffer H (20 mM HEPES, pH 7.9; 15 mM MgCl_2_; 10 mM KCl; 1 mM DTT; 1 X Complete protease inhibitor) for 30 min. Heart samples was transferred to a Dounce homogenizer (2 ml Kontes Glass Co, Vineland, NJ) and dissociated by 10 strokes with the loose fitting pestle A and incubated on ice for 5 min. Further dissociation was carried out by 10 strokes with tight fitting pestle B, performed every 5 min for 15 min. Nuclei were collected by centrifugation (2000 × *g*, 4 °C) and re-suspended in 1 ml of nuclei pulldown buffer NPB buffer (10 mM HEPES, pH 7.9; 40 mM NaCl; 90 mM KCl; 0.5 mM EDTA; 0.5 mM spermidine; 0.15 mM spermine; 1 mM dithiothreitol and 1 X Complete protease inhibitor). For nuclei purification, nuclei were incubated with 250 µg of M-280 streptavidin-coated dynabeads (Invitrogen) with rotation for 30 min at 4 °C. A flow-based system was used to capture the nuclei bound on the streptavidin beads. A 10 ml seriological pipette (VWR) attached to a 1 ml micropipette tip (Rainin reach pipet tip), both pre-treated with NPB+1% BSA) for 30 min, was added to a MiniMACS separator magnet (OctoMACS Separator, Miltenyl Biotec). A two-way stopcock (Biorad) was connected to the end of the 1 ml micropipette tip via a piece of Tygon tubing (Fisher Scientific) and the flow-rate set to ~0.75 ml/min. The nuclei beads suspension was diluted by addition of 9 ml of NPBt (NPB with 0.01% Triton X-100) and added to the slow-flow setup. The tip was subsequently removed from the stand and the nuclei-beads released from the tip by slowly pipetting 1 ml of NPBt in and out of the tip. The solution was then diluted again to 10 ml with NPBt and added again to the slow-flow setup. Nuclei-beads were eluted in 1 ml of NPBt as described above and the NPBt removed using a magnetic stand (DynaMag TM-2 magnet, Invitrogen). Nuclei-beads were then processed for RNA extraction.

### Mouse flow cytometry & gating

Single cell cardiac tissue suspensions were prepared by mincing hearts and gentle agitation in collagenase 500 units per ml in HBSS solution for 1 h at 37 °C. Samples were passed through a 70 μm filter, washed and resuspended in 1% FBS/PBS. Samples were incubated with FcR-block, and then labelled with anti-CD45 (Biolegend 103124, 1:200), anti-CD11b (Biolegend 101206, 1:100), anti-Ly6G (Biolegend 127636, 1:200), anti-F4/80 (Biolegend 123110, 1:100), anti-Ly6C (Biolegend 128026 1:800), anti-CD206 (Biolegend 141721, 1:100). Where indicated, macrophages were sorted on a FACSAriaIII. Cell populations were gated as shown in Supplementary Fig. 2. Briefly, doublets were excluded (by FSC-W vs FSC-A) and dead cells removed by 7-AAD. Myeloid cells were gated for CD45^+^, CD11b^+^, and neutrophils identified by positivity for Ly6G. Macrophages were identified as Ly6G F4/80^+^ cells. Monocytes were identified as Ly6G F4/80^+^ LyC^hi/lo^ cells. Analysis was performed using FlowJo v10.0.8.

### RNA extraction and library preparation

Zebrafish total nuclear RNA extraction and DNAse treatment were carried out using the RNAqueous Micro Kit (Life Technologies) according to manufacturer’s instructions. RNA integrity was checked with a RNA pico chip (Agilent Technologies) using the Agilent 2100 Bioanalyzer. cDNA was synthesized and amplified from 100 pg–300 pg of input RNA using SMART-seq^TM^v4 Ultra Low input RNA kit (Clontech laboratories). Sequencing libraries were prepared using the Nextera XT DNA library preparation kit.

Mouse RNA was extracted from FAC-sorted macrophages using the RNAqueous®-Micro Total RNA isolation kit from Life Technologies (Ambion). cDNA was produced using the Clontech SMART-Seq v4 Ultra Low Input RNA kit, and libraries constructed with the Nextera XT DNA Sample Preparation kit, according to the manufacturers’ instructions. Libraries were quantified using an Agilent Bioanalyzer with High Sensitivity DNA analysis kit, pooled and run on a Nextseq 2500.

### RNA-seq analysis

Next Generation Sequencing was performed on a NextSeq500 platform using a NextSeqTM500 150-cycle High Output Kit) (Illumina) to generate 80-basepair paired end reads. Read quality was evaluated using FastQC^59^. Zebrafish reads were mapped to the Jul. 2014 Zv10/danRer10 assembly version of the zebrafish genome using STAR (v.2.4.2a) splice-aware aligner^60^. Count tables were generated using subread FeatureCounts (v1.4.5-p1q), with standard parameters^61^. Differential expression was carried out using DESeq2 R package^62^. Raw and processed data generated in this study were submitted to GEO (accession number GSE100029), differential expression outputs are available as Supplementary Data File 1. Hierarchical clustering was performed on genes significantly differentially expressed in at least one comparison (*p*-value<0.05). Comparisons where *p*-values were not available were excluded. log_2_ Fold Change values from DESeq2 analysis were used to calculate Euclidian distances, and Ward’s minimum variance method (Ward D) was used to perform hierarchical clustering (k = 9). For mouse, trimmed reads (Trim Galore v.0.3.7, http://www.bioinformatics.babraham.ac.uk/projects/trim_galore, with default settings) were screened for quality (FastQC v.011.3, http://www.bioinformatics.bbsrc.ac.uk/projects/fastqc) and then mapped to the mouse genome (Mm10) using TopHat^63^ v2.1.10, with Bowtie^64^ v.2.2.6, using the “b2-sensitive” setting with mate inner distance = 208 and mate standard deviation = 213 determined from test alignments. Transcriptome annotations downloaded from the Illumina iGenomes website (UCSC Mm10, https://support.illumina.com/sequencing/sequencing_software/igenome.html) were used with Cufflinks for transcript mapping. Cuffdiff was used for pair-wise comparisons. Genes with log_2_ fold change > 2 and FDR < 0.05 were deemed significantly differentially expressed. Cuffdiff results were loaded into R (http://www.R-project.org) for further analysis and plotting, including Principal components analysis (PCA) using the pcaMethods package^65^ and heatmap generation with pheatmap (http://CRAN.R-project.org/package=pheatmap). For temporal analyses, raw counts from Cuffdiff were retrieved and genes with < 5 counts in all samples were removed before carrying out tests. DESeq2^62^ was used to identify genes showing differential temporal regulation and STEM v.1.3.8^66^ was used for temporal clustering and gene ontology enrichment analysis of temporal profiles. Raw and processed data generated in this study were submitted to GEO (accession number GSE126772) and differential expression outputs are available as Supplementary Data File 2. A custom R script (Supplementary Data File 3) was used to generate the cluster overlap significance plot of Supplementary Fig. 5.

### sgRNA, Cas9 mRNA synthesis and injections

sgRNA template DNA was generated with a unique oligonucleotide encoding the T7 RNA polymerase recognition site, the sgRNA target sequence and an overlap with the tracrRNA. The full list of gene-specific oligonucleotides are available in Supplementary Table 3. Each gene-specific sgRNA oligo was first annealed with the universal reverse oligo containing tracrRNA sequence (AAAAGCACCGACTCGGTGCCACTTTTTCAAGTTGATAACGGACTAGCCTTATTTTAACTTGCTATTTCTAGCTCTAAAAC) and subsequently amplified by PCR. In vitro transcription was performed with 500–1000 ng purified DNA template for using the T7 RNA polymerase kit (NEB) for 4 h at 37 °C, and sgRNA purified using the Megaclear kit (Ambion).

Cas9 mRNA was in vitro transcribed from plasmid MLM3613 (Addgene, cat # 42251) using the mMESSAGE mMACHINE T7 kit (Ambion). sgRNA’s (20 pg/nl) and cas9 mRNA (90 pg/nl) were injected at the single cell stage.

### HRMA analysis

Genomic DNA was extracted from single embryos at 24 hpf using the Purelink Genomic DNA minikit (Invitrogen). Primers were designed to generate a ~200 bp product spanning the cut site, Supplementary Table 4. Hotshot Diamond PCR mastermix (Client Lifescience) was used to perform PCR with LC Green Plus dye (BioFire Diagnostics). Reaction solutions were cycled on a C1000 Touch^TM^ Bio-Rad thermal cycler.

### Next Generation Sequencing to validate genome editing events

Gene-specific primers with illumina i5 and i7 adaptors were designed to amplify the predicted Cas9 cleavage site generating an amplicon of ~120 bp in size. In a second round of amplification, libraries were made and indexed using the Nextera XT Index kit (Illumina). Libraries were quantified by Qubit ds DNA HS Assay kit (Thermo Fisher Scientific, Q32854) and the size verified by Tapestation D1000 (Agilent). Libraries were sequenced with a MiSeq 300 cycles reagent kit (Illumina).

### Embryonic “trio” tagging of endogenous collagen proteins

We combined homology independent repair-based genome editing with intron-based protein trapping (“trio” tagging) in order to endogenously tag *col4a1* in the zebrafish embryo. Introns were selected based on regions of the protein where insertion of a tag would not disturb collagen polymerisation, its export into the extracellular matrix or generation of functional fibrils^67,68^. The successfully tagged intron was flanked by exons 23 and 24. 1 nl of the injection solution containing two sgRNAs targeting the donor vector in the region harbouring a Citrine exon flanked by splice acceptor and donor sites^69^ and one sgRNA targeting the intronic region of the gene of interest (each at 12.5 ng/uL), the donor construct (25 ng/uL; full sequence of the FlipTrap vector available through NCBI accession no. JN564735^69^), Cas9 protein (100 ng/uL; PNA Bio Inc) and 10% phenol-red (5 mg/mL in 150 mM KCl; Sigma-Aldrich) was injected into *Tg(mpeg1:mCherry)*^*gl23*^ embryos at the 1-cell stage. For sgRNAs sequences, please check Supplementary Table 3. For HRMA primers, please check Supplementary Table 4. All components of the “trio” tagging were pre-assembled 24 h in advance of injection and stored at −20 °C, as this was shown to increase efficiency of integration of the donor vector. After the CRISPR/Cas9-mediated knock-in of the linearised donor fragment into the targeted intron of *col4a1* in vivo, a fusion mRNA of *Citrine* flanked by exons 23 and 24 of *col4a1* was generated, similar to the approach recently reported in vitro^70^. Embryos were monitored, assessed for fluorescence and collected for FACS at 6 dpf. mCherry + macrophages were sorted and used for adoptive transfer experiments. Approximately 30–40% of the injected embryos showed mosaic labelling of the protein of interest. The domain of expression of the Citrine-fused protein phenocopied the reported expression pattern deposited in the Zfin database (https://zfin.org). For efficient tagging of the endogenous protein to occur, all components of the “trio” tagging solution needed to be present. No collagen-Citrine expression was observed when the intron-targeting sgRNA was omitted from the pre-assembled injection solution.

### Fluorescence activated cell sorting (FACS) and adoptive cell transfer studies

Zebrafish GFP^+^ macrophages were isolated from *Tg(mpeg1:EGFP)*^*gl22*^ operated adult hearts or whole embryos by FACS. mCherry^+^ macrophages were isolated from *Tg(mpeg1:mCherry)*^*gl23*^ “trio”-tagged whole embryos by FACS. Neural crest cells used as control in adoptive cell transfer experiments were isolated from 16-somite stage *Gt(foxd3-citrine)*^*ct110*^ embryos. Prior to FACS, tissue was dissociated using 20 mg/ml collagenase in 0.05% Trypsin/0.53 mM EDTA/1xHBSS buffer to obtain single cell suspensions. Reaction was stopped in 10 mM HEPES/0.25% BSA/1xHBSS buffer and GFP^+^ and/or mCherry^+^ cells were sorted (FACSAria, BD Biosciences Fusion System). Sorted cells were spun down at low speed, re-suspended in 5μl Hanks buffer and immediately used for transplantation. Adoptive transfer of macrophages (approximately 3,000 cells if adult-derived; 20,000–30,000 cells if obtained from embryos) into recipient animals was performed by retro-orbital injection^39^ of 5μl of cell suspension or Hanks buffer. 6, 13 and 20 days after transplantation, zebrafish were sacrificed and hearts were harvested.

Mouse monocytes were isolated from the spleen of CD68-GFP^+^ and GFP*tpz*-collagen adult mice. Following schedule 1 killing, the spleen was removed by dissection and the tissue disrupted with the blunt end of a sterile syringe. The disrupted spleen tissue was washed through a 70 μm cell filter into red cell lysis buffer and incubated at room temperature for 10 min. Cells were then spun down (400 × *g* for 5 min) and red cell lysis buffer removed by aspiration. Monocytes were then purified from the cell pellet using the EasySep^TM^ Mouse Monocyte Enrichment Kit according to the manufacturer’s instructions. Prior to surgery, i.e. permanent ligation of the LAD, cells were counted and resuspended in sterile PBS. For neonatal adoptive transfer experiments, each recipient animal (postnatal day 1; P1) received 50000 cells by intra-myocardial injection (using a 30G insulin syringe) at the time of LAD ligation, whereas for adult adoptive transfer experiments, each recipient animal received 100,000 cells intra-myocardial injection (using a 30G insulin syringe) at the time of MI surgery.

### Isolation of cardiac cells

Following MI, mouse hearts were harvested for flow cytometry studies at day 7 post-injury; individual hearts were isolated, placed in cold HBSS (Life Technologies), finely minced into small pieces, and digested with collagenase type II (Worthington Laboratories) solution (containing 500 units/ml HBSS) at 37 °C for 30 min with agitation. Supernatant was removed and 10% heat-inactivated FBS (Sigma) added. The remaining tissue was digested with a fresh collagenase solution for a total of 3 times. Cell suspensions were combined and filtered through a 40μm cell strainer (BD Falcon). Cells were centrifuged, washed with PBS, and Red Cell Lysis buffer (BioLegend) used according to the manufacturer’s instructions to remove red blood cells. Isolated single cardiac cells were stained and subjected to flow cytometric analyses.

### Flow cytometry of GFP+ cell within infarcted hearts

For flow cytometry studies *Col1a1-GFP* transgenic mice^37^ that underwent LAD ligation were harvested and processed as described above. Isolated cells were resuspended in 2% FBS/PBS solution and blocked with incubation in FcBlock on ice. The macrophage population was identified by immunostaining for 20 min at room temperature using an antibody F4/80-PE (BioLegend Inc., catalogue number 123110, 1:100 dilution). For fibroblast staining on processed heart samples, isolated cells were stained with anti-Feeder Cells-APC (Miltenyl Biotec clone mEF-SK4, 1:20). The 7-aminoactinomycin D high fluorescence dye (7AAD) was added prior to cell analyses to determine cell viability and exclude dead cells. Flow cytometric analyses were performed using a BDFACSAriaIII flow cytometer (BD Biosciences) and the FlowJo software.

### Bone marrow-derived macrophage (BMDM) isolation and culture

Bone marrow cells were isolated from femurs and tibias of 3–5 months old GFP*tpz*-collagen transgenic mice. Cells were plated on 100 mm non-tissue culture treated petri dish (Thermo Fisher, UK) and maintained in macrophage differentiation media (DMEM supplemented with 10% heat-inactivated fetal bovine serum (FBS), 2 mM L-glutamine, 2% penicillin/streptomycin and 20% supernatant from L929 cells as a source of macrophage colony-stimulating factor). On day 7 post isolation, BMDMs were positively selected by trypsinization and subsequent wash-out of non-adherent cells. After positive selection, BMDMs were maintained in macrophage-growing media (DMEM supplemented with 10% FBS, 2 mM L-glutamine and 2% penicillin/streptomycin) for 1–2 days before being plated for imaging.

### Stimulation of GFP-collagen deposition

GFP*tpz*-collagen BMDMs were seeded on matrigel-coated coverslips at a cell density of 3 × 10^4^ cells/cm^2^ together with murine L929 cells (1.5 × 10^4^ cells/cm^2^, obtained from the American Type Culture Collection). In order to stimulate collagen deposition, growing medium (DMEM with 10% FBS, 2 mM L-glutamine and 2% penicillin/streptomycin) was supplemented with a combination of supernatants (SNTs) from stimulated L929 fibroblasts and Mouse primary Cardiac microvascular Endothelial Cells, MCEC, (C57-6024, obtained from Cell Biologics) in a ratio of 2:1:1 (growing medium:L929 SNT:MCEC SNT) during 6 to 9 days. To facilitate collagen deposition, 50 µg/ml of fresh ascorbic acid (A92902, Sigma-Aldrich) was added daily and medium supplemented with SNTs was changed every 2 days. For supernatants preparation, L929 and MCEC monoculture were grown to confluence in DMEM supplemented with 10% FBS, 2 mM L-glutamine and 2% penicillin/streptomycin. L929 cells medium was supplemented with 10 ng/ml TNFα (410-MT/CF, R&D Systems), 10 ng/ml IL-1β (401-ML/CF, R&D Systems), 10 ng/ml IL-4 (404-ML/CF, R&D Systems) and 10 ng/ml TGFβ (7666-MB/CF, R&D Systems). MCEC medium was supplemented with 10 ng/ml TNFα (410-MT/CF, R&D Systems). After 48 h of incubation, the supernatants from both monocultures were harvested, centrifuged to remove cell debris and stored at −20 °C until use.

### Immunofluorescence of BMDM-fibroblast co-cultures

Cultures were stimulated with L929 SNT in a 1:1 ratio with growing medium. L929 SNT was obtained from confluent L929 stimulated with 10 ng/ml TNFα and 10 ng/ml IL-1β during 6 days. Cells were fixed in 4% paraformaldehyde but no permeabilization step was performed in order to preserve extracellular collagen structure. Cells were incubated with protein block serum free solution (X0909, Dako) during 1 h at room temperature and with primary antibodies overnight at 4 °C. Antibodies used were anti-CD68 (Abcam) 1:250, anti-GFP (Abcam) 1:500 and anti-Col1a (Abcam) 1:200. Cells were mounted in ProLong™ Diamond mounting medium with DAPI (Thermo Fisher). Images were acquired using a Leica DM600 CFS confocal microscope.

### Collagen fibre quantification

To quantify collagen deposition, images were analysed using the ImageJ plug-in NeuronJ^71^. Briefly, images were divided into 4 quadrants and GFP*t*pz^+^ collagen fibres length was measured in each quadrant using the NeuronJ tracing tool. Total length of collagen fibres per quadrants containing 0 or 1 macrophage was plotted against collagen fibre length measured in quadrants containing more than one macrophage. Associations between collagen fibre length and macrophage content were tested using 2-tailed, unpaired Student’s *t*-test. Values were considered significant at *p < 0.05.

### Immunofluorescence, in situ HCR and imaging

For immunostaining of sections, zebrafish fixed hearts were embedded in OCT, cryosectioned and stained^8^. Briefly, non-specific binding sites were saturated by incubation for 1 h in blocking solution (5% goat serum, 0.3% Tween-20). Primary antibodies used were anti-mpeg1 (1:200, GeneTex), MF20 anti-myosin heavy chain (1:200, DSHB), anti-mCherry (1:200, Clontech; 1:150, GeneTex), anti-Collagen I (1:100, Abcam), and anti-GFP (1:200, Abcam). Alexa (405, 488, 555, 633/647)-conjugated secondary antibodies (1:1000, Invitrogen) were used to reveal primary antibody signal. Nuclei were stained with Hoechst/DAPI and slides were mounted in Vectashield (Vector). Imaging was performed on a Zeiss 780 Upright MP confocal microscope. For HCR (version 3), fixed hearts were wax-embedded and 7μm sections deparaffinised, rehydrated and washed in DEPC-treated water. Staining was performed as previously described^72^. Briefly, sections were pre-hybridised with probe hybridization buffer for 10 min at 37 °C and then incubated with 0.4 pmol of each DNA probe (*mpeg*, *col4a1* and *col4a3bpa*; Molecular Instruments) diluted in hybridization buffer, covered with parafilm and incubated overnight at 37 °C in a humidified chamber. Sections were washed in probe wash buffer and incubated for 30 min at room temperature in amplification buffer. Hairpins were incubated at final concentration of 6 pmol each (amplifier B3-Alexa594, amplifier B4-Alexa488 and amplifier B5-Alexa647; Molecular Instruments), overnight at room temperature in a dark humidified chamber. Excess hairpins were washed in 5× Sodium Chloride Sodium Citrate/0.1% Tween 20. Sections were imaged on a Zeiss 780 Upright MP confocal microscope. For imaging endogenous “trio” tagged collagen, *Tg(mpeg1:mCherry)*^*gl23*^ embryos at 6 dpf were mounted in 1% low-melting agarose. Sequential Z-stack series were obtained with a Zeiss 780 Upright MP confocal microscope. Citrine signal was excited with a 514 nm laser and mCherry with 561 nm laser. For whole-mount immunostaining, fixed hearts were bleached for 1 h in 15% hydrogen peroxide/PBS, washed in PBS containing 0.5% Triton-X and blocked in 2% BSA overnight at 4 °C. Anti-GFP (1:50, Abcam) primary antibody was incubated overnight at 4 °C, washed at length in PBS containing 0.1% Triton-X. Alexa 488-conjugated secondary antibody (1:200, Invitrogen) was incubated overnight at 4 °C and washed. Nuclei were stained with Hoechst. Hearts were mounted in 1% low melting point agarose (Sigma) and imaged on a Zeiss Z1 Lightsheet microscope.

Mouse hearts were fixed overnight in 4% PFA at 4 °C and prepared for cryosectioning. Sections were processed for indirect immunofluorescence using standard methods. Primary antibodies used were rabbit anti-Collagen I (Abcam ab34710, 1:100 dilution), rat anti-CD68 (Bio-Rad MCA1957, 1:100 dilution), chicken anti-GFP (Abcam ab13970, 1:100). AlexaFluor secondary antibodies (Invitrogen, 1:200) were used in all cases. Imaging was performed using an Olympus FV1000 confocal microscope. Images were digitally captured and processed using either ImageJ or FIJI software (NIH Image, Bethesda, MD).

### Histology and scar measurements

Fixed zebrafish hearts were wax-embedded and 7 μm sections deparaffinised, rehydrated and washed in distilled water. Acid Fuchsin Orange-G (AFOG) and Masson’s trichrome staining were performed^6,73^. For morphometric analysis, 6 representative sections of the whole heart were imaged per sample using an Axio Scope.A1 polarized light microscope fitted with an AxioCam HR camera. The scar region and the ventricular surface were demarcated and areas measured using ImageJ software. The percentage of the scar size relative to the entire ventricle was calculated.

Mouse hearts were collected, fixed in 4% PFA overnight and either stored in PBS, or embedded in paraffin wax. 10 μm paraffin sections were stained by Masson’s trichrome (Abcam) according to the manufacturer’s protocol. All images were processed using ImageJ software.

### Statistical analysis

For zebrafish, scar measurements were done blindly. Graphs and statistical analyses were generated by Graphpad prism, version 7. Results are expressed as the mean ± SEM. We used a 2-tailed, unpaired Student’s *t*-test for all pair-wise comparisons. Not significant, *p* > 0.05; *p < 0.05; **p < 0.003; ***p < 0.0001. For mouse, statistical difference between groups was evaluated using 2-tailed, unpaired Student’s *t*-test or one-way ANOVA. A *p*-value of < 0.05 was considered statistically significant. All values and graphs present the mean value ± SEM.

### Reporting summary

Further information on research design is available in the Nature Research Reporting Summary linked to this article.

## Supplementary information

Supplementary Information

Description of Additional Supplementary Files

Supplementary Data 1

Supplementary Data 2

Supplementary Data 3

Reporting Summary

## Data Availability

Raw and processed data generated in this study were submitted to GEO with the following accession numbers: zebrafish GSE100029 and mouse GSE126772. Differential expression outputs are available as Supplementary Data File 1 and Supplementary Data File 2, respectively. Custom R scripts used to generate the cluster overlap significance plot of Supplementary Data Fig. 5 are available as Supplementary Data File 3. Source data for Figs. 4–6 and Supplementary Figs. 1, 2, 6, 7 and 9 are provided with the paper.

## Source Data

Source Data
