# Supplementary material for: Macrophages directly contribute collagen to scar formation during zebrafish heart regeneration and mouse heart repair
Source: Nat Commun. 2020 Jan 30;11:600. doi: 10.1038/s41467-019-14263-2 (PMC6992796; doi:10.1038/s41467-019-14263-2)
Supplement: Supplementary file 1 — Supplementary Information [file 41467_2019_14263_MOESM1_ESM.pdf]

Supplementary Information

Macrophages directly contribute collagen to scar formation during zebrafish heart regeneration and mouse heart repair

Simões, et al.

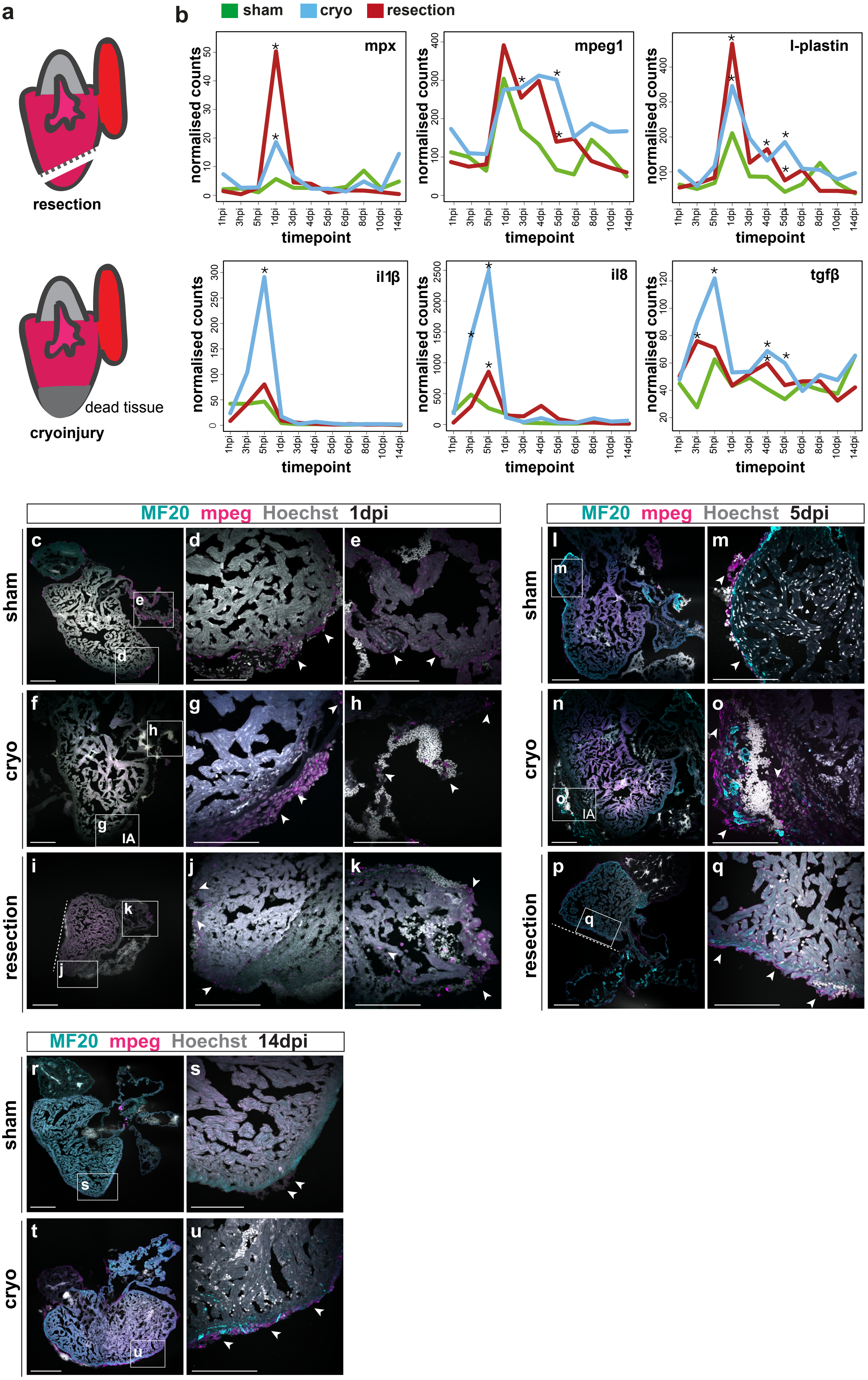
a
b
sham
cryo
resection
resection
cryoinjury
dead tissue
mpx
mpeg1
l-plastin
il1β
il8
tgfβ
normalised counts
timepoint
1hpi
3hpi
5hpi
1dpi
3dpi
4dpi
5dpi
6dpi
8dpi
10dpi
14dpi
MF20 mpeg Hoechst 1dpi
MF20 mpeg Hoechst 5dpi
MF20 mpeg Hoechst 14dpi
sham
cryo
resection
IA

Supplementary Figure 1. Molecular and cellular immune responses to injury during cardiac regeneration in zebrafish. (a) Zebrafish heart regeneration injury models: ventricular resection and ventricular cryoinjury. (b) Time-course expression profiles for induced genes in sham- (green), cryo- (blue), and resection- (red) operated hearts at different stages post-injury. *Mpx*, mpeg, *l-plastin*, *il1β*, *il8* and *tgfβ* normalized transcript counts (Y axis), generated by Nanostring nCounter analysis, R plotted across time (X axis). Data represent the mean, *$p<0.05$ (n=3 hearts per time-point, one-way ANOVA). (c-u), Confocal imaging of heart sections stained with mpeg (red) to visualize macrophages in c, l, r sham- f, n, t, cryo- and i, p, resection- operated zebrafish hearts at c-k, 1 dpi (respective white boxes enlarged in d, g, j for ventricle close-up and e, h, k for atrium detail), l-q 5 dpi (corresponding white boxes enlarged in m, o, q for ventricle detail), and r-u 14 dpi (matching white boxes enlarged in s, u for ventricle detail). Myocardium is labelled with MF20 (cyan) and nuclei are visualized with Hoechst (grey). Arrowheads point to mpeg+ macrophages, dashed line represents plane of amputation in resectioned hearts and IA with a cryoprobe. Representative images of n=3 per group. Scale bar: 200μm (insets showing high-magnification images, scale bar: 100μm); hpi, hours post injury; dpi, days post injury; IA, injury area. Source data are provided as a Source Data file.

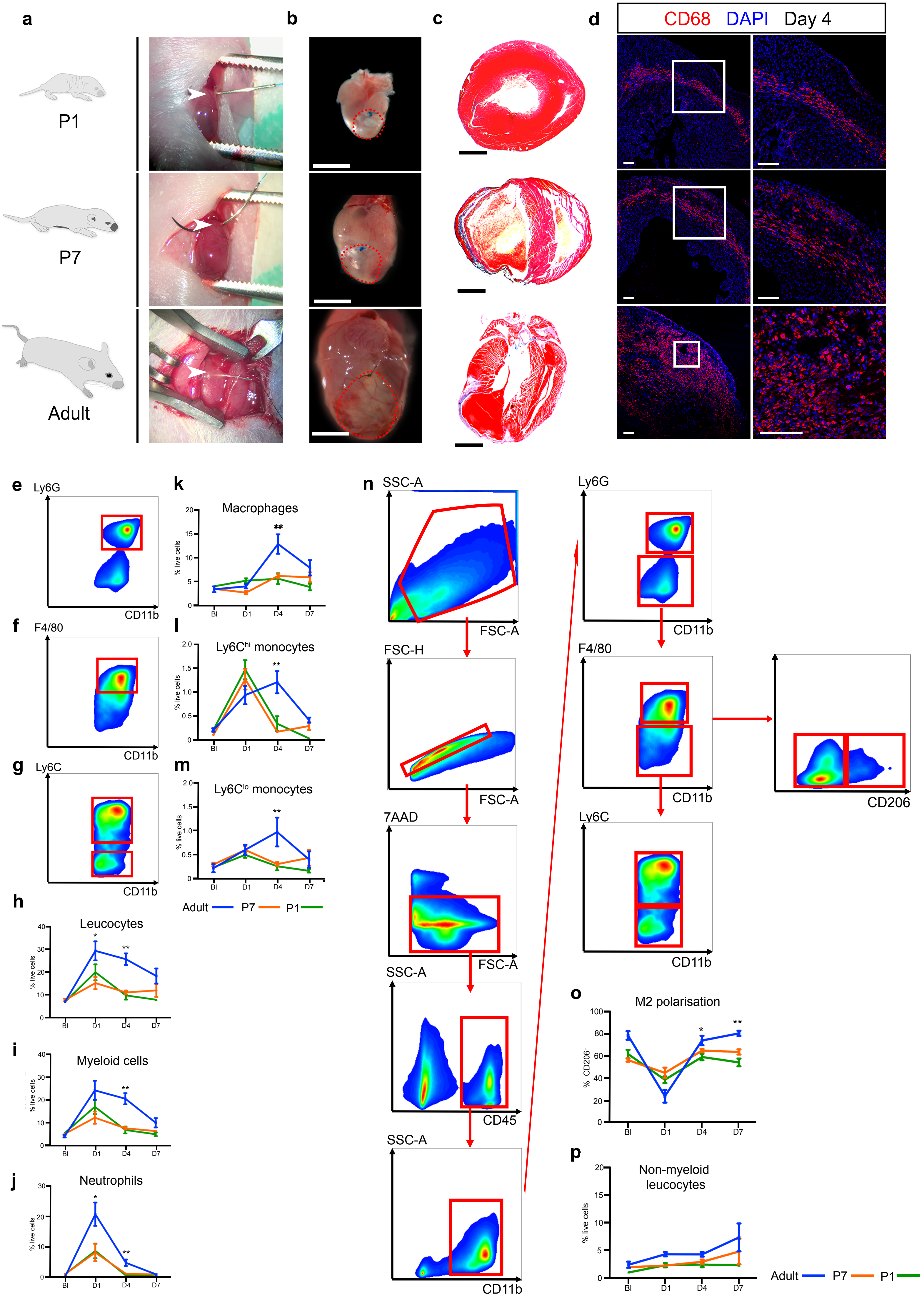

a
b
c
d
P1
P7
Adult
CD68 DAPI Day 4
e
Ly6G
CD11b
f
F4/80
CD11b
g
Ly6C
CD11b
h
Leucocytes
% live cells
Bl D1 D4 D7
i
Myeloid cells
% live cells
j
Neutrophils
% live cells
k
Macrophages
% live cells
l
Ly6C^hi monocytes
% live cells
m
Ly6C^lo monocytes
% live cells
Adult P7 P1
n
SSC-A
FSC-A
FSC-H
FSC-A
7AAD
FSC-A
SSC-A
CD45
SSC-A
CD11b
Ly6G
CD11b
F4/80
CD11b
CD206
Ly6C
CD11b
o
M2 polarisation
% CD206+
p
Non-myeloid leucocytes
% live cells
Adult P7 P1

Supplementary Figure 2. Immune responses to injury during murine neonatal cardiac regeneration and adult cardiac repair. (a) Surgeon's view of mouse myocardial infarction (MI) procedure in the postnatal day 1 (P1) neonatal mouse, the postnatal day 7 (P7) neonatal mouse, and the adult mouse heart. The position of the left anterior descending coronary artery is highlighted (arrowhead). (b) Macroscopic view of the P1, P7 and adult mouse heart at day 4 following MI surgery, demonstrating tissue injury (highlighted in red) downstream of coronary artery ligation. Scale bar, 2mm. (c) Masson's trichrome-stained sections from the P1, P7 and adult mouse heart at day 21 post-MI, demonstrating regeneration in the P1, in comparison to extensive myocardial loss and scar formation in the P7 and adult heart. Scale bar, 2mm. (d) Confocal imaging of immunofluorescence stained heart cryosections demonstrating localisation of macrophages ($CD68^+$ cells, red) the infarct zone in the anterior ventricular wall in the P1, P7 and adult mouse (representative of n=3 per group). Respective white boxes enlarged in right hand panel. Scale bar, 100 μm. (e-g) Gating for flow cytometric identification of neutrophils ($CD45^+$ $CD11b^+$ $Ly6G^+$), macrophages ($CD45^+$ $CD11b^+$ $Ly6G^-$ $F4/80^+$) and monocytes ($CD45^+$ $CD11b^+$ $Ly6G^-$ $F4/80^-$ $Ly6C^{hi/lo}$) in the adult heart post-MI; flow cytometry images shown are at day 1 post-MI; positive gate indicated by red box). (h-m) Quantification of leucocytes (h), myeloid cells (i), neutrophils (j), macrophages (k), $Ly6C^{hi}$ monocytes (l), and $Ly6C^{lo}$ monocytes (m) in the adult (blue), P7 (orange) and P1 (green) heart following MI. (n) full gating strategy: flow cytometry images shown are at day 1 post-MI; positive gate indicated by red box. (o,p) Quantification of macrophage polarisation (o), and non-myeloid leucocytes (p) in the adult (blue), P7 (orange) and P1 (green) heart following MI. Cell numbers are expressed as a proportion of total live cells. Data represent the mean ± SEM *$p<0.05$, **$p<0.01$, one-way ANOVA. Source data are provided as a Source Data file.

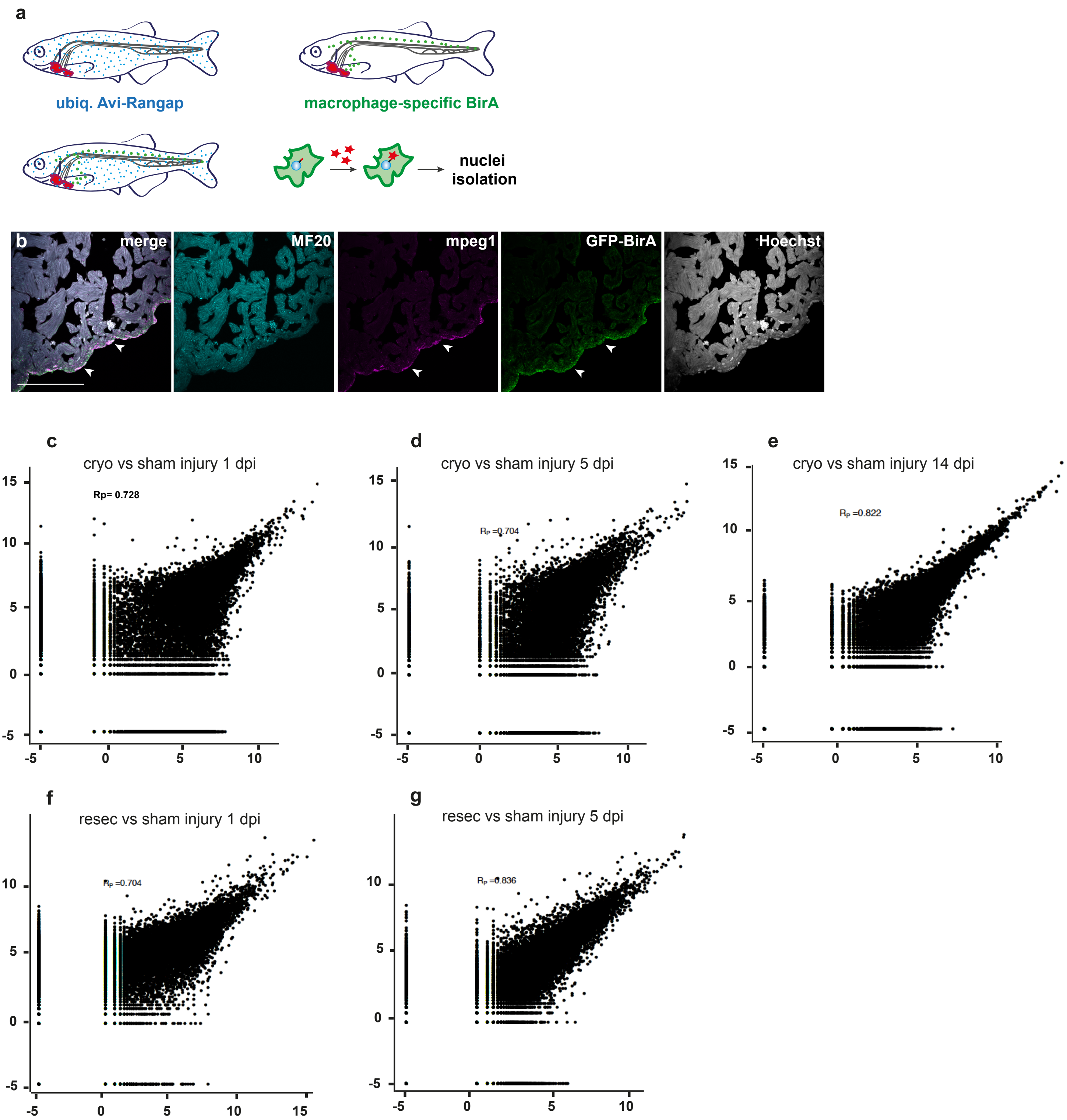

a
ubiq. Avi-Rangap
macrophage-specific BirA
nuclei isolation
b
merge
MF20
mpeg1
GFP-BirA
Hoechst
c
cryo vs sham injury 1 dpi
Rp= 0.728
d
cryo vs sham injury 5 dpi
Rp =0.704
e
cryo vs sham injury 14 dpi
Rp =0.822
f
resec vs sham injury 1 dpi
Rp =0.704
g
resec vs sham injury 5 dpi
Rp =0.836

Supplementary Figure 3. Biotagging of macrophage nuclei and technical reproducibility of replicates. (a) Schematic of binary transgenic zebrafish model for regulatory profiling of macrophages. Biotagging effector transgenic zebrafish line ubiquitously expressing Avi-tagged Rangap for biotinylation of nuclei crossed to biotagging driver line expressing BirA in macrophage-specific manner, generating a double positive zebrafish embryo, raised to adulthood, which will have macrophage nuclei biotinylated and ready to be isolated. Red stars represent biotin. (b) Confocal imaging of *TgBAC(mpeg1:BirA-Citrine)*^{ox122} adult heart sections stained with mpeg (fuschia) and GFP (green) to visualize transgene expression in macrophages (white arrowheads). Myocardium is labelled with MF20 (cyan) and nuclei with Hoechst (grey). Representative images of n=3 per group. Scale bar: 100μm. (c-g), scatterplot of $\log_{10}$ (normalised counts) between biological duplicates for representative macrophage nuclear samples of (c) 1 dpi cryo 1 vs 1 dpi sham 2; (d) 5 dpi cryo 2 vs 5 dpi sham 2; (e) 14 dpi cryo 4 vs 14 dpi sham 4; (f) 1 dpi resec 1 vs 1 dpi sham 3; and (g) 5 dpi resec 1 vs 5 dpi sham 2.

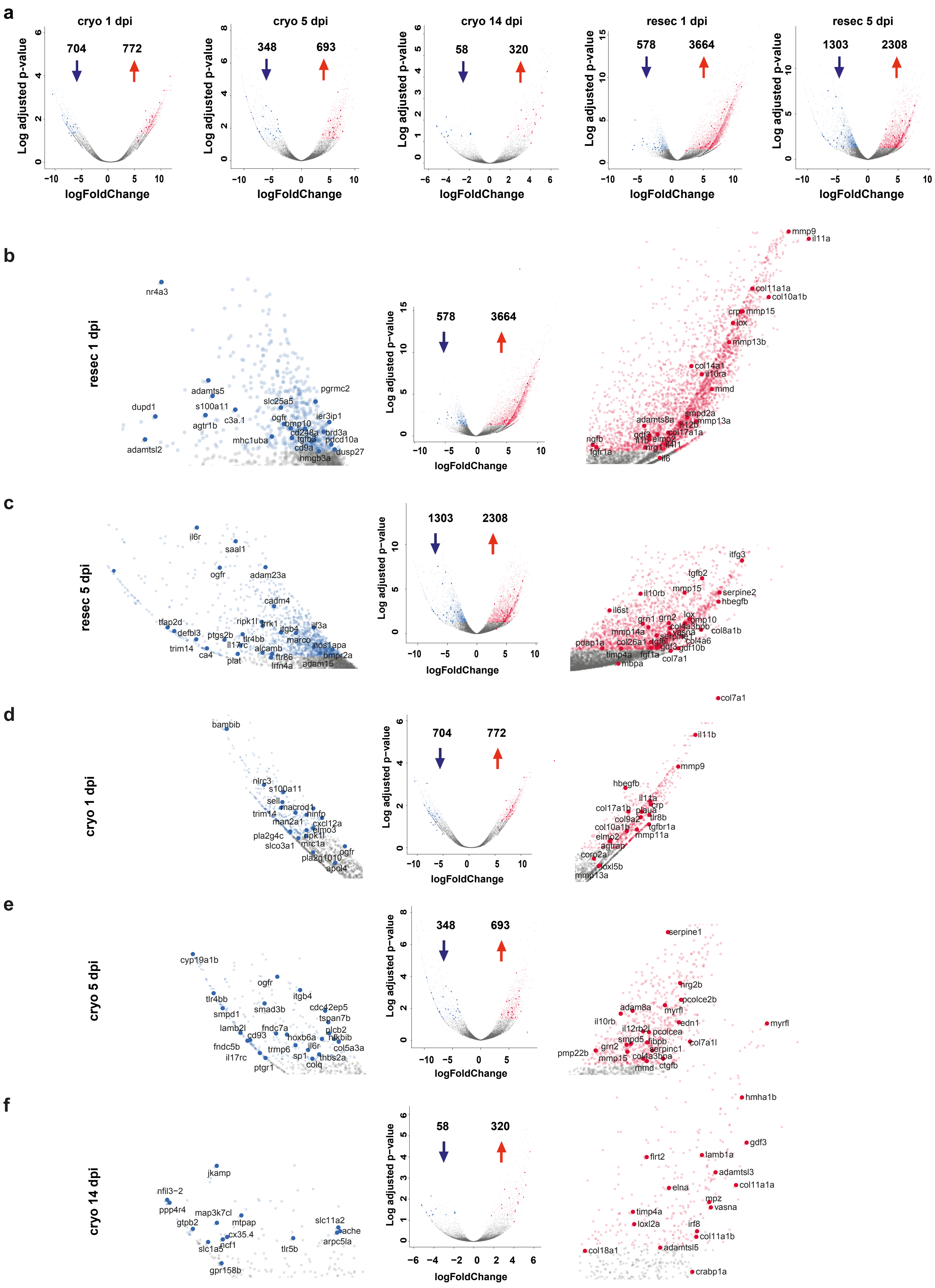
a
cryo 1 dpi
704
772
cryo 5 dpi
348
693
cryo 14 dpi
58
320
resec 1 dpi
578
3664
resec 5 dpi
1303
2308
Log adjusted p-value
logFoldChange
b
resec 1 dpi
nr4a3
adamts5
dupd1
s100a11
agtr1b
c3a.1
adamtsl2
mhc1uba
slc25a5
pgrmc2
ogfr
bmp10
ier3ip1
cd248a
brd3a
tgfb3
pdcd10a
cd9a
dusp27
hmgb3a
mmp9
il11a
col11a1a
col10a1b
crp
mmp15
lox
mmp13b
col14a1
il10ra
mmd
smpd2a
adamts8a
mmp13a
il12b
gdf3
col17a1a
il1b
elmo2
ngfb
nrg1
il4l1
fgfr1a
il6
c
resec 5 dpi
il6r
saal1
ogfr
adam23a
cadm4
tfap2d
ripk1l
defbl3
trk1
itgb4
ilf3a
ptgs2b
tlr4bb
marco
trim14
il17rc
nos1apa
ca4
alcamb
plat
ftr86
bmpr2a
lrfn4a
adam15
itfg3
tgfb2
il10rb
mmp15
serpine2
hbegfb
il6st
lox
grn1
grn2
bmp10
col4a3bpb
mmp14a
vasna
col8a1b
serpinf
pdap1a
col26a1
tgfbi
col4a6
gdf3
gdf10b
timp4a
fgf1a
col7a1
mbpa
d
cryo 1 dpi
bambib
nlrc3
s100a11
sell
macrod1
trim14
hinfp
man2a1
cxcl12a
elmo3
pla2g4c
ripk1l
slco3a1
mrc1a
ogfr
pla2g1010
apol4
col7a1
il11b
mmp9
hbegfb
il11a
crp
col17a1b
plaua
col9a2
tlr8b
col10a1b
tgfbr1a
elmo2
mmp11a
agtrap
coro2a
loxl5b
mmp13a
e
cryo 5 dpi
cyp19a1b
ogfr
itgb4
tlr4bb
smad3b
cdc42ep5
smpd1
tspan7b
lamb2l
fndc7a
plcb2
cd93
hoxb6a
nfkbib
fndc5b
il6r
col5a3a
trmp6
il17rc
sp1
thbs2a
colq
ptgr1
serpine1
nrg2b
pcolce2b
adam8a
myrfl
il10rb
edn1
il12rb2l
pcolcea
smpd5
fibpb
col7a1l
grn2
serpinc1
pmp22b
mmp15
col4a3bpa
mmd
ctgfb
f
cryo 14 dpi
jkamp
nfil3−2
ppp4r4
map3k7cl
mtpap
gtpb2
cx35.4
slc11a2
ache
slc1a5
ncf1
tlr5b
arpc5la
gpr158b
hmha1b
gdf3
flrt2
lamb1a
adamtsl3
col11a1a
elna
mpz
vasna
timp4a
irf8
loxl2a
col11a1b
col18a1
adamtsl5
crabp1a

Supplementary Figure 4. Volcano plots of differentially expressed genes in zebrafish macrophages. (a) Volcano plots show differential expression analysis for injury models vs sham control transcripts as the relationship between p-value and log fold change (red-upregulated; blue-downregulated) for cryo-injury (1 dpi), cryo-injury (5 dpi), cryo-injury (14 dpi), resec-injury (1 dpi), resec-injury (5 dpi). (b-f) Volcano plot of DESEq2-generated differential expression analysis in cryo and resection injury models vs sham controls shows the relationship between p-value and log fold change (red-upregulated; blue-downregulated; grey-not significantly differentially expressed) for (b) 1 dpi resection, (c) 5 dpi resection, (d) 1 dpi cryo, (e) 5 dpi cryo and (f) 14 dpi cryo.

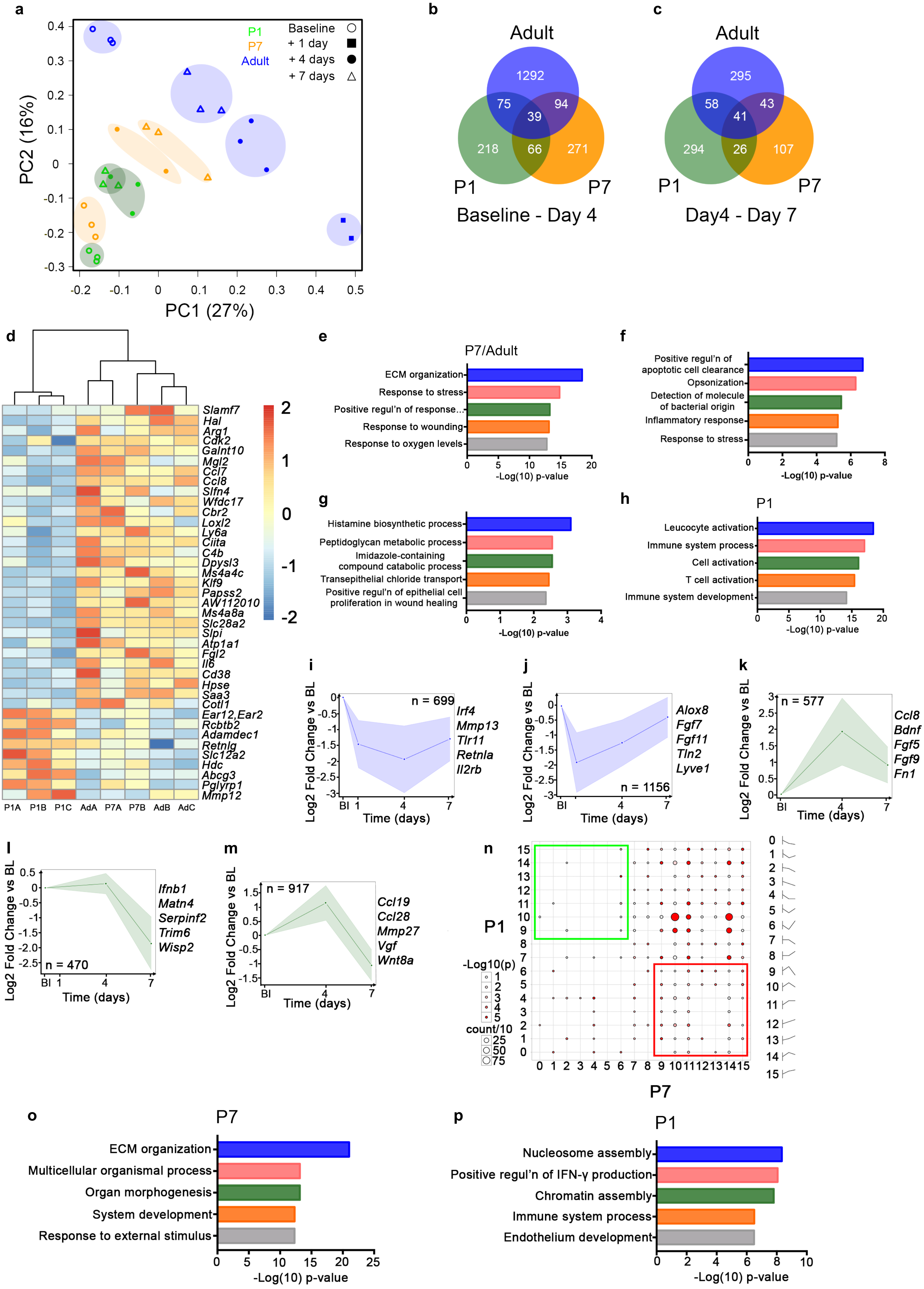
a
P1
P7
Adult
Baseline
+ 1 day
+ 4 days
+ 7 days
PC2 (16%)
PC1 (27%)
b
Adult
1292
75
94
39
218
66
271
P1
P7
Baseline - Day 4
c
Adult
295
58
43
41
294
26
107
P1
P7
Day4 - Day 7
d
Slamf7
Hal
Arg1
Cdk2
Galnt10
Mgl2
Ccl7
Ccl8
Slfn4
Wfdc17
Cbr2
Loxl2
Ly6a
Ciita
C4b
Dpysl3
Ms4a4c
Klf9
Papss2
AW112010
Ms4a8a
Slc28a2
Slpi
Atp1a1
Fgl2
Il6
Cd38
Hpse
Saa3
Cotl1
Ear12,Ear2
Rcbtb2
Adamdec1
Retnlg
Slc12a2
Hdc
Abcg3
Pglyrp1
Mmp12
P1A P1B P1C AdA P7A P7B AdB AdC
e
P7/Adult
ECM organization
Response to stress
Positive regul'n of response...
Response to wounding
Response to oxygen levels
-Log(10) p-value
f
Positive regul'n of apoptotic cell clearance
Opsonization
Detection of molecule of bacterial origin
Inflammatory response
Response to stress
-Log(10) p-value
g
Histamine biosynthetic process
Peptidoglycan metabolic process
Imidazole-containing compound catabolic process
Transepithelial chloride transport
Positive regul'n of epithelial cell proliferation in wound healing
-Log(10) p-value
h
P1
Leucocyte activation
Immune system process
Cell activation
T cell activation
Immune system development
-Log(10) p-value
i
n = 699
Irf4
Mmp13
Tlr11
Retnla
Il2rb
Log2 Fold Change vs BL
Time (days)
j
n = 1156
Alox8
Fgf7
Fgf11
Tln2
Lyve1
Log2 Fold Change vs BL
Time (days)
k
n = 577
Ccl8
Bdnf
Fgf5
Fgf9
Fn1
Log2 Fold Change vs BL
Time (days)
l
n = 470
Ifnb1
Matn4
Serpinf2
Trim6
Wisp2
Log2 Fold Change vs BL
Time (days)
m
n = 917
Ccl19
Ccl28
Mmp27
Vgf
Wnt8a
Log2 Fold Change vs BL
Time (days)
n
P1
P7
-Log10(p)
count/10
o
P7
ECM organization
Multicellular organismal process
Organ morphogenesis
System development
Response to external stimulus
-Log(10) p-value
p
P1
Nucleosome assembly
Positive regul'n of IFN-γ production
Chromatin assembly
Immune system process
Endothelium development
-Log(10) p-value

Supplementary Figure 5. Transcriptomic analysis of macrophages in regenerative and scar-forming models of mouse myocardial infarction. (a) Principal Component Analysis showing differential gene expression between macrophages from the P1 (green), P7 (orange) and adult (blue) mouse heart at baseline and indicated time points (day 1, 4 and 7) following MI. Biological replicates are highlighted. (b,c) Venn diagrams demonstrating the number of differentially expressed genes in macrophages from P1 (green), P7 (orange) and adult (blue) hearts between baseline and day 4 (left panel), and day 4 and day 7 (right panel). Common genes are shown in overlapping segments. (d) Heatmap showing semi-hierarchical clustering of differentially expressed transcripts between regenerative (P1) and scar-forming (P7 and adult, Ad) mouse models at day 4 post-MI. Red - high expression. Yellow - medium expression. Blue - low expression. Gene names are indicated. (e) Gene ontology (GO) terms associated with differentially expressed genes in the P7 and adult mouse (vs P1) at day 7 post-MI. (f) Gene ontology (GO) terms associated with differentially expressed genes in the P7 and adult mouse (vs P1) at day 4 post-MI. (g) GO terms associated with differentially expressed genes in the P1 mouse (vs P7/adult) at day 4 post-MI. (h) GO terms associated with differentially expressed genes in the P1 mouse (vs P7/adult) at day 7 post-MI. (i,j) Unbiased temporal clustering demonstrating dynamically regulated gene sets in macrophages across the phases of scar-based wound healing in the adult mouse heart post-MI (n = number of genes within set). Significant profiles of genes downregulated in inflammation (i) and in the transition from injury to repair (j) are shown, with selected genes highlighted. (k-m) Unbiased temporal clustering demonstrating dynamically regulated gene sets in macrophages across the time period of regenerative healing in the P1 neonatal mouse heart post-MI. Significant profiles of gene sets transiently upregulated (k) or downregulated (l,m) in regeneration are shown (n=number of genes within set). Selected genes are highlighted. (n) Heatmap showing size of gene overlap (circle size) and statistical significance (colour) between all macrophage temporal profiles in the P1-regenerating and P7-scar forming mouse heart. Profile shape is shown in the legend. Discordantly

moving genes are highlighted in green (initially upwardly expressed in P1, downwardly expressed in P7) and red (initially upwardly expressed in P7, downwardly expressed in P1). (o) GO terms associated with divergently upregulated genes across time in macrophages in the P7 mouse (vs P1). (p) GO terms associated with divergently upregulated genes across time in macrophages in the P1 mouse (vs P7).

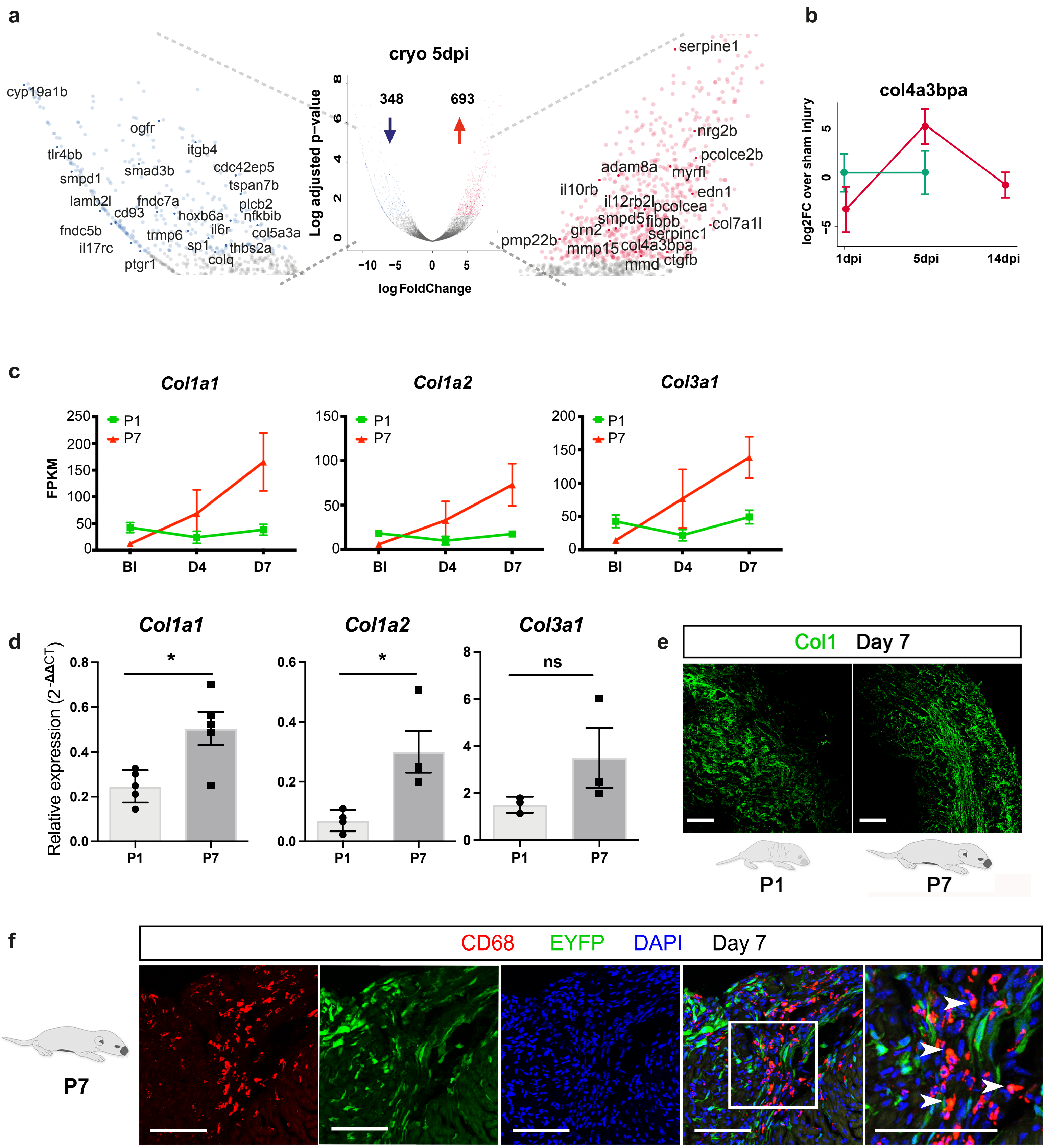
a
b
cryo 5dpi
348
693
Log adjusted p−value
log FoldChange
cyp19a1b
ogfr
tlr4bb
itgb4
cdc42ep5
smad3b
smpd1
tspan7b
lamb2l
fndc7a
plcb2
cd93
hoxb6a
nfkbib
fndc5b
il6r
col5a3a
trmp6
il17rc
sp1
thbs2a
colq
ptgr1
serpine1
nrg2b
pcolce2b
adam8a
myrfl
il10rb
edn1
il12rb2l
pcolcea
smpd5
fibpb
col7a1l
grn2
serpinc1
pmp22b
mmp15
col4a3bpa
mmd
ctgfb
col4a3bpa
log2FC over sham injury
1dpi
5dpi
14dpi
c
Col1a1
Col1a2
Col3a1
P1
P7
FPKM
BI
D4
D7
d
Col1a1
Col1a2
Col3a1
Relative expression (2-ΔΔCT)
*
*
ns
P1
P7
e
Col1 Day 7
P1
P7
f
CD68 EYFP DAPI Day 7
P7

Supplementary Figure 6. Expression of collagens in macrophages. (a) Volcano plots show differential expression analysis for zebrafish injury models vs sham control transcripts as the relationship between p-value and log fold change (693-upregulated (red); 348-downregulated (blue)) for cryoinjury at 5 dpi. (b) Graph of $\log_2$ fold change shows col4a3bpa upregulation at 5 dpi in cryoinjury model. (c) RNA sequencing demonstrating that macrophages dynamically upregulate the fibrillar collagen genes *Col1a1*, *Col1a2* and *Col3a1* during the wound healing phase post-MI in the mouse heart. FPKM, Fragments Per Kilobase of transcript per Million mapped reads. (d) qPCR showing upregulation of fibrillar collagen genes *Col1a1*, *Col1a2*, *Col3a1* in whole heart samples from the P7 mouse compared to the P1 at day 7 post-MI. (e) Confocal imaging of immunofluorescence-stained heart cryosections at day 7 post-MI from P1 and P7 mice, demonstrating disorganised expression of collagen I (green) across the injury site in the anterior left ventricular wall in the P1 mouse, in contrast to organised, fibrillar collagen I deposition in the P7 mouse (representative of n=3 per group, scale bar: 100μm). (f) Confocal imaging of immunofluorescence-stained heart cryosections at day 7 post-MI from Col1a2-CreERT2;R26R-EYFP mice. Tamoxifen activation of *Cre* recombinase was performed at day 5 post-MI. $CD68^+$ macrophages (red) are seen to express YFP confirming expression at the Col1a2 promoter (representative of n=3, scale bar: 100μm). Source data are provided as a Source Data file.

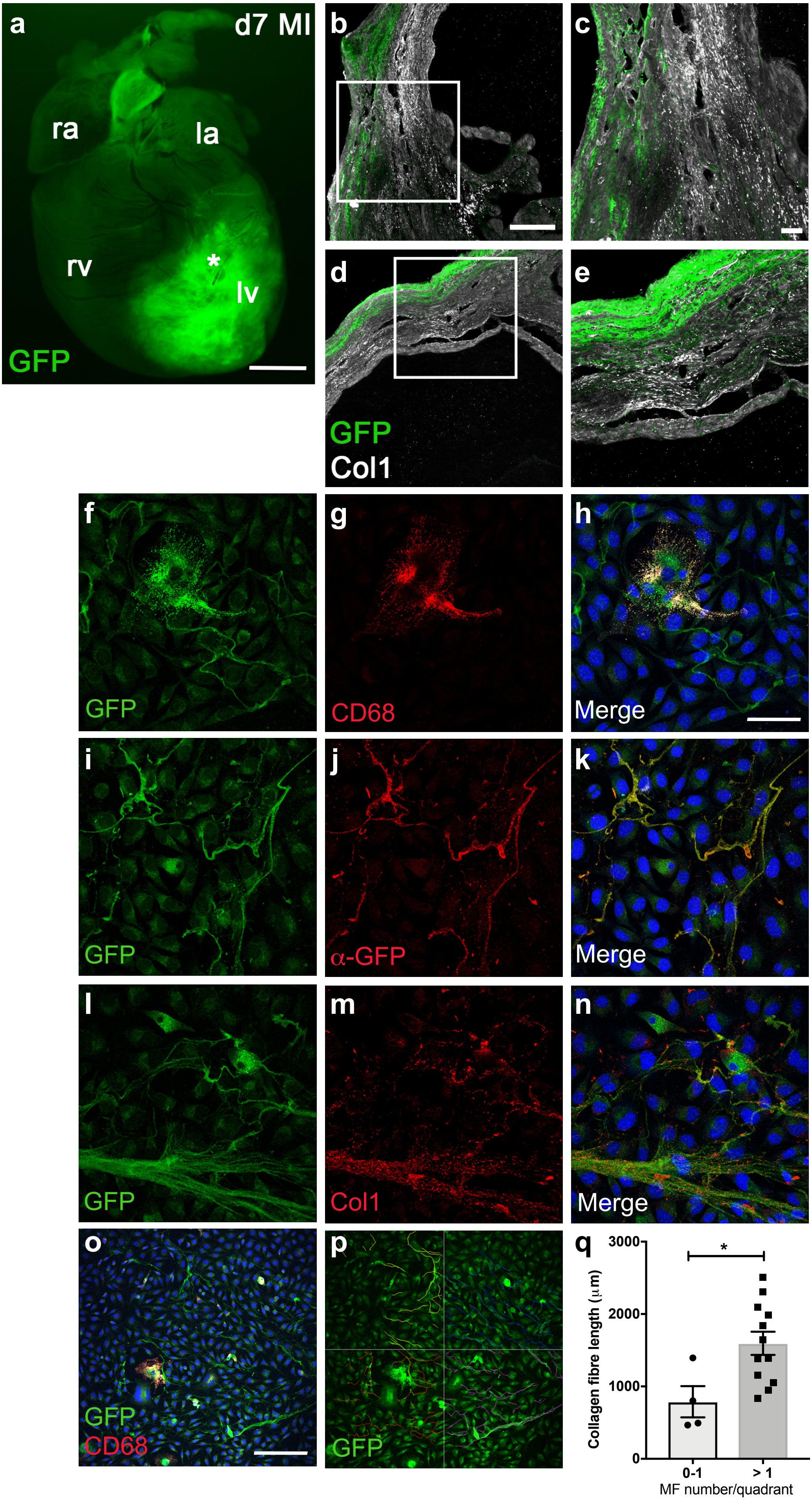
a
d7 MI
ra
la
rv
lv
GFP
b
c
d
e
GFP
Col1
f
GFP
g
CD68
h
Merge
i
GFP
j
α-GFP
k
Merge
l
GFP
m
Col1
n
Merge
o
GFP
CD68
p
GFP
q
Collagen fibre length (μm)
3000
2000
1000
0
0-1
> 1
MF number/quadrant

Supplementary Figure 7. Characterisation of the scar post-MI in adult GFP*tpz*-collagen mice and deposition of GFP*tpz*-collagen *in vitro* by activated bone marrow-derived macrophages. (a) Whole mount view of the GFP+ scar region in left ventricle of GFP*tpz*-collagen 1 heart at day 7 post-MI; suture location is indicated by a white asterisk. (b-e) Cryo-sections revealed GFP and Col1 co-staining in regions of the left ventricle proximal to the infarct (white inset boxes in left hand panels magnified in the right hand panels). la- left atrium , ra – right atrium, lv-left ventricle, rv- right ventricle, pm- proximal myocardium to the infarct/scar, rm- remote myocardium. Scale bars in A 1mm, in b (as applies to d) 100μm and c (as applies to e) 50μm. (f-h) Confocal imaging of GFP*tpz*-collagen+ bone marrow-derived monocytes (BMDM) co-cultured with L929 fibroblasts and an activating cytokine cocktail (TNF-α, Il-1β, IL4 and TGFβ) revealed GFP*tpz*+ (green) fibres around CD68 (red) positive macrophages (i-k) GFP*tpz*+ (green) fibres colocalized with anti-GFP immunostaining excluding autofluorescence and (l-n) with anti-Col1 immunostaining confirming collagen fibril identity. Scale bar in h (as relates to f-n) is 50μm. (o,p) Collagen fibre deposition was quantified from images divided in to 4 quadrants and fibres were traced using the ImageJ plugin NeuronJ; (q) total fibre length per quadrant containing 0 or 1 macrophage (0-1) or more than one macrophage (>1) revealed a significant increase in fibre length with increased incidence of macrophages in culture. Data represent the mean ± SEM of 4 cultures per group (n=4). *$p<0.05$; scale bar in o (as applies to p) is 100μm. Source data are provided as a Source Data file.

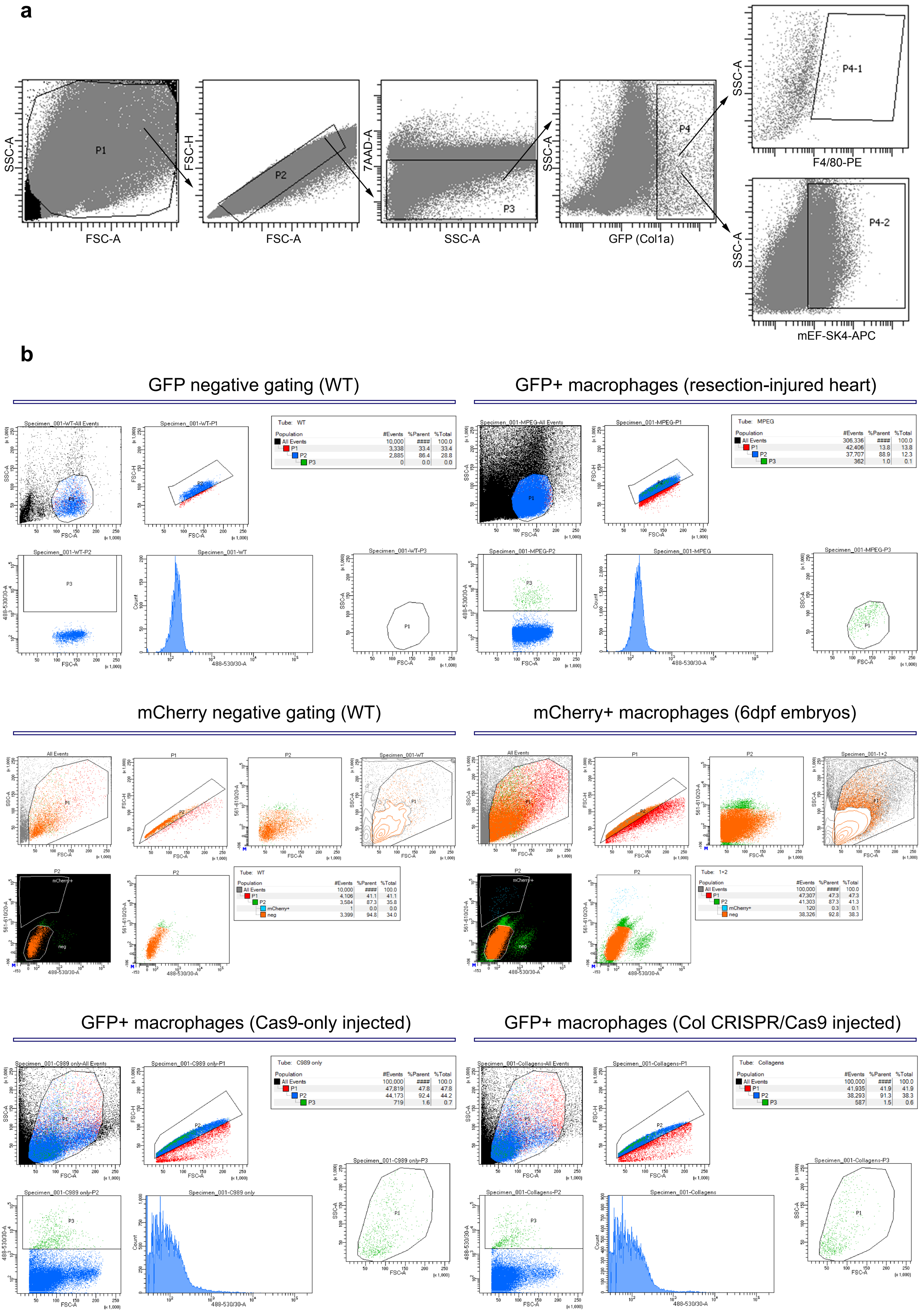
a
SSC-A
FSC-A
P1
FSC-H
P2
7AAD-A
SSC-A
P3
GFP (Col1a)
P4
F4/80-PE
P4-1
mEF-SK4-APC
P4-2
b
GFP negative gating (WT)
GFP+ macrophages (resection-injured heart)
mCherry negative gating (WT)
mCherry+ macrophages (6dpf embryos)
GFP+ macrophages (Cas9-only injected)
GFP+ macrophages (Col CRISPR/Cas9 injected)
Tube: WT
Population #Events %Parent %Total
All Events 10,000 #### 100.0
P1 3,338 33.4 33.4
P2 2,885 86.4 28.8
P3 0 0.0 0.0
Tube: MPEG
Population #Events %Parent %Total
All Events 306,336 #### 100.0
P1 42,406 13.8 13.8
P2 37,707 88.9 12.3
P3 362 1.0 0.1
Tube: WT
Population #Events %Parent %Total
All Events 10,000 #### 100.0
P1 4,106 41.1 41.1
P2 3,584 87.3 35.8
mCherry+ 1 0.0 0.0
neg 3,399 94.8 34.0
Tube: 1+2
Population #Events %Parent %Total
All Events 100,000 #### 100.0
P1 47,307 47.3 47.3
P2 41,303 87.3 41.3
mCherry+ 120 0.3 0.1
neg 38,326 92.8 38.3
Tube: C989 only
Population #Events %Parent %Total
All Events 100,000 #### 100.0
P1 47,819 47.8 47.8
P2 44,173 92.4 44.2
P3 719 1.6 0.7
Tube: Collagens
Population #Events %Parent %Total
All Events 100,000 #### 100.0
P1 41,935 41.9 41.9
P2 38,293 91.3 38.3
P3 587 1.5 0.6
488-530/30-A
561-610/20-A
Count

Supplementary Figure 8. Gating strategies used for cell sorting. (a) Gating strategy to analyse the contribution of collagen 1-alpha by cardiac macrophages and fibroblasts following MI. The initial population is a single-cell suspension derived from individual Col1a-GFP+ adult hearts (n=5). This gating strategy refers to data in Supplementary Table 2. (b) Gating strategy used to sort *Tg(mpeg1:EGFP)*^{gls22} ventricular resected injured zebrafish hearts (refers to data in Figure 5a-h and Supplementary Figure 9a-d). Gating strategy used in adoptive cell transfer experiments of macrophages expressing fluorescent Citrine-tagged endogenous *col4a1* generated by CRISPR/Cas9-mediated intron-based protein trapping in (*Tg(mpeg1:mCherry)*^{gl23} 6dpf (days post fertilization) transgenic embryos. Macrophages were solely sorted based on their mCherry+ expression. This gating strategy refers to data in Figure 5i-r and Supplementary Figure 9e. Gating strategy used during adoptive cell transfer experiments using macrophages carrying genetic deletions of *col4a3bpa* and *col4a1*. Transgenic *Tg(mpeg1:EGFP)*^{gl22} zebrafish embryos were injected with *Cas9* mRNA only (Cas9-only injected) or with Cas9 mRNA combined with gRNAs targeting both *col4a3bpa* and *col4a1* genes (Col CRISPR/Cas9 injected) and then FAC-sorted. This gating strategy refers to data in Figure 6 and Supplementary Figure 9k-m. The same gating strategy was used to data presented in Supplementary Figure 9h-j. For all zebrafish experiments, WT-derived (fluorescent negative) cells were used as an initial gate to remove any autofluorescent cells from our experimental conditions.

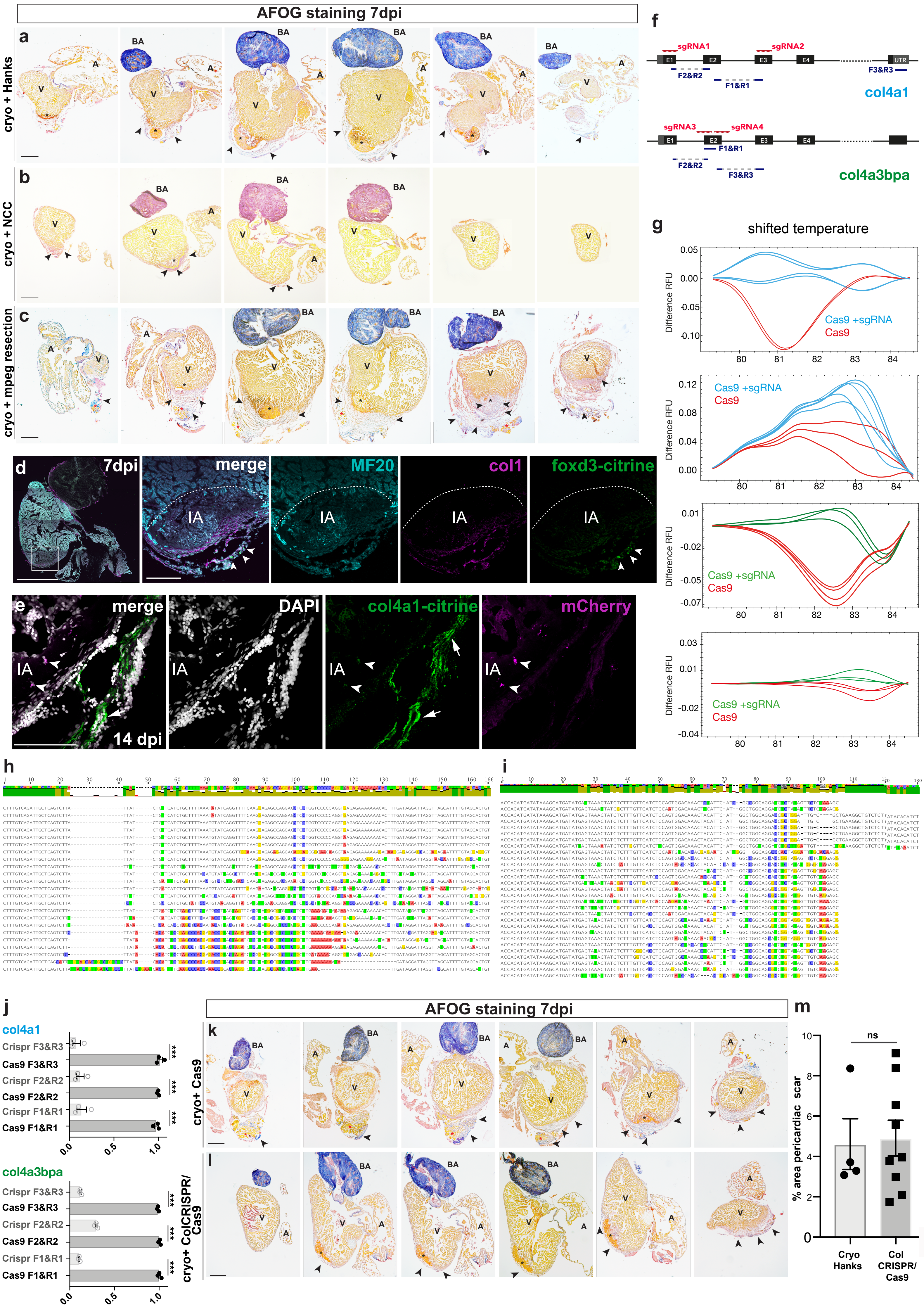
AFOG staining 7dpi
a
cryo + Hanks
b
cryo + NCC
c
cryo + mpeg resection
BA
A
V
d
7dpi
merge
MF20
col1
foxd3-citrine
IA
e
merge
DAPI
col4a1-citrine
mCherry
14 dpi
f
sgRNA1
sgRNA2
E1
E2
E3
E4
UTR
F2&R2
F1&R1
F3&R3
col4a1
sgRNA3
sgRNA4
col4a3bpa
g
shifted temperature
Difference RFU
Cas9 +sgRNA
Cas9
h
i
j
col4a1
Crispr F3&R3
Cas9 F3&R3
Crispr F2&R2
Cas9 F2&R2
Crispr F1&R1
Cas9 F1&R1
col4a3bpa
***
AFOG staining 7dpi
k
cryo+ Cas9
l
cryo+ ColCRISPR/Cas9
m
ns
% area pericardiac scar
Cryo Hanks
Col CRISPR/ Cas9

Supplementary Figure 9. Transplanted macrophages can deposit collagen during zebrafish heart repair regeneration. (a-c) Acid Fuchsin Orange-G (AFOG) staining of serial heart sections (6 sections per heart) at the onset of scar formation shows healthy myocardium (yellow), injured myocardium (orange) and collagen (blue). More fibrotic tissue (arrowheads) at the periphery of the cryo-injured area (black asterisk) is observed in hearts transplanted with resection injury-derived GFP+ macrophages (c), when compared to control Hanks-injected (a) and neural crest cells transplanted (b) control hearts. Scale bar: 200μm; A, atrium; V, ventricle; BA, Bulbus Arteriosus; red asterisk, myocardial dead tissue detached from the injury region. Quantification details in Figure 5h. (d) Antibody staining of cryo-injured WT hearts transplanted with neural crest cells derived from 16-somite *Gt(foxd3-citrine)*^{ct110} embryos show citrine+ cells (arrowheads) near the collagen1-expressing (fuschia) injury region (IA, demarked by dotted line) 7 days after injury. Representative images of n=3 per group. Scale bar d: 200μm; high-magnification inset 100μm. (e) Confocal imaging of 14dpi WT recipient hearts transplanted with col4a1 "trio"-citrine tagged mCherry macrophages. Antibody staining reveals a mosaic extracellular "green" fibrillar scar (arrows). Intracellular collagen-citrine staining is observed in the few mCherry-transplanted macrophages that are still present in the injury area (IA) at 14dpi/13dp transplantation (arrowheads). Scale bar: 100μm. (f) Representation of sgRNAs (in red) targeting *col4a1* (blue) and *col4a3bpa* (green) and the relative location of each respective primer pairs (F1&F2; F2&R2 and F3&R3) used for qPCR quantification. (g) HRMA temperature-shifted difference curves showing the melting profile of re-annealed amplicons generated from a single embryo injected with Cas9 only (red lines) or co-injected with Cas9+col4a1 sgRNA (blue lines) or Cas9+col4a3bpa sgRNA (green lines). Relative Fluorescence Units (RFU). (h,i) representative alignment over two targeted regions (h, *col4a1*; i, *col4a3bpa*) clearly reflects clonal structure of CRISPR/Cas9-induced indels in GFP-negative cells sorted from *Tg(mpeg1:EGFP)*^{gl22} embryos injected with Cas9 only or *col4a3bpa* plus *col4a1* sgRNAs/Cas9. (j) qPCR quantification of *col4a1* and *col4a3bpa* transcript levels

in macrophages FAC-sorted from 6dpf *Tg(mpeg1:EGFP)*^{gl22} embryos injected with Cas9 only or *col4a3bpa* plus *col4a1* sgRNAs/Cas9. Three different primer pairs per target gene (details of targeted regions in f) were used to quantify the downregulation of both transcripts in the F_0-edited macrophages, in comparison to Cas9-only injected controls (*col4a3bpa:* F1&R1 Cas9: 1.005 ± 0.013, n=3 vs CRISPR/Cas9: 0.106 ± 0.006, n=3, ***$p<0.001$; F2&R2 Cas9: 1.007 ± 0.008, n=3 vs CRISPR/Cas9: 0.296 ± 0.009, n=3, ***$p<0.001$; F3&R3 Cas9: 0.998 ± 0.005, n=3 vs CRISPR/Cas9: 0.121 ± 0.007, n=3, ***$p<0.001$. *Col4a1:* F1&R1 Cas9: 0.978 ± 0.021, n=3 vs CRISPR/Cas9: 0.137 ± 0.055, n=3, ***$p<0.001$; F2&R2 Cas9: 0.999 ± 0.006, n=3 vs CRISPR/Cas9: 0.122 ± 0.043, n=3, ***$p<0.001$; F3&R3 Cas9: 1.021 ± 0.027, n=3 vs CRISPR/Cas9: 0.079 ± 0.043, n=3, ***$p<0.001$); data are mean ± SEM; 2-tailed, unpaired Student's t-test used for pair-wise comparisons. (k, l) Acid Fuchsin Orange-G (AFOG) staining of serial heart sections (6 sections per heart) showing healthy myocardium (yellow), injured myocardium (orange) and collagen (blue). (k) excess scar tissue at the periphery of the wound (arrowheads) stained in hearts transplanted with 6 days post fertilization (dpf) GFP+ macrophages injected with Cas9 only. (l) hearts transplanted with *col4a1+col4a3bpa* CRISPR/Cas9 macrophages show reduced collagen staining at the periphery of the wound. Scale bar: 200μm; A, atrium; V, ventricle; BA, Bulbus Arteriosus; black asterisk, cryo-injured area; red asterisk, myocardial dead tissue detached from the injury region. (m) quantification as a percentage of total section area (6 sections per heart) show no significant difference in scar deposition in the group of cryo-injured hearts transplanted with *col4a1+col4a3bpa* CRISPR/Cas9 macrophages and control Hanks-injected (Cryo+Hanks: 4.61 ± 1.26%, n=4 vs Cryo+Col CRISPR/Cas9: 4.91 ± 0.88%, n=9; $p=0.854$, ns, not significant; data are mean ± SEM, 2-tailed, unpaired Student's t-test).). Data are mean ± SEM. ns, not significant. Source data are provided as a Source Data file.

	1dpi_sham1	1dpi_sham2	1dpi_sham3	1dpi_cryo1	1dpi_cryo2	5dpi_sham2	5dpi_sham3	5dpi_sham1	5dpi_cryo1	5dpi_cryo2	14dpi_sham1	14dpi_sham2	14dpi_sham3	14dpi_sham4	14dpi_cryo4	14dpi_cryo1	14dpi_cryo2	14dpi_cryo3
**1dpi_sham1**	1	0.786	0.795	0.771	0.818	0.827	0.825	0.771	0.769	0.809	0.798	0.829	0.832	0.819	0.821	0.801	0.815	0.814
**1dpi_sham2**	0.786	1	0.775	0.751	0.803	0.813	0.817	0.732	0.748	0.790	0.772	0.814	0.817	0.806	0.808	0.793	0.795	0.811
**1dpi_sham3**	0.795	0.775	1	0.757	0.800	0.802	0.796	0.742	0.752	0.773	0.765	0.809	0.814	0.804	0.795	0.793	0.793	0.791
**1dpi_cryo1**	0.771	0.751	0.757	1	0.818	0.770	0.784	0.714	0.733	0.771	0.754	0.784	0.785	0.773	0.778	0.768	0.771	0.772
**1dpi_cryo2**	0.818	0.803	0.800	0.818	1	0.843	0.852	0.764	0.771	0.832	0.812	0.847	0.849	0.833	0.845	0.817	0.835	0.838
**5dpi_sham2**	0.827	0.813	0.802	0.770	0.843	1	0.943	0.817	0.797	0.874	0.855	0.916	0.923	0.887	0.925	0.855	0.897	0.907
**5dpi_sham3**	0.825	0.817	0.796	0.784	0.852	0.943	1	0.813	0.799	0.882	0.854	0.910	0.913	0.878	0.925	0.856	0.894	0.913
**5dpi_sham1**	0.771	0.732	0.742	0.714	0.764	0.817	0.813	1	0.746	0.796	0.769	0.810	0.811	0.791	0.809	0.782	0.804	0.800
**5dpi_cryo1**	0.769	0.748	0.752	0.733	0.771	0.797	0.799	0.746	1	0.780	0.774	0.805	0.802	0.794	0.796	0.776	0.787	0.790
**5dpi_cryo2**	0.809	0.790	0.773	0.771	0.832	0.874	0.882	0.796	0.780	1	0.816	0.863	0.864	0.844	0.876	0.835	0.859	0.871
**14dpi_sham1**	0.798	0.772	0.765	0.754	0.812	0.855	0.854	0.769	0.774	0.816	1	0.867	0.866	0.838	0.865	0.811	0.848	0.848
**14dpi_sham2**	0.829	0.814	0.809	0.784	0.847	0.916	0.910	0.810	0.805	0.863	0.867	1	0.921	0.889	0.916	0.856	0.895	0.905
**14dpi_sham3**	0.832	0.817	0.814	0.785	0.849	0.923	0.913	0.811	0.802	0.864	0.866	0.921	1	0.890	0.918	0.862	0.900	0.907
**14dpi_sham4**	0.819	0.806	0.804	0.773	0.833	0.887	0.878	0.791	0.794	0.844	0.838	0.889	0.890	1	0.883	0.842	0.865	0.876
**14dpi_cryo4**	0.821	0.808	0.795	0.778	0.845	0.925	0.925	0.809	0.796	0.876	0.865	0.916	0.918	0.883	1	0.860	0.898	0.918
**14dpi_cryo1**	0.801	0.793	0.793	0.768	0.817	0.855	0.856	0.782	0.776	0.835	0.811	0.856	0.862	0.842	0.860	1	0.846	0.855
**14dpi_cryo2**	0.815	0.795	0.793	0.771	0.835	0.897	0.894	0.804	0.787	0.859	0.848	0.895	0.900	0.865	0.898	0.846	1	0.890
**14dpi_cryo3**	0.814	0.811	0.791	0.772	0.838	0.907	0.913	0.800	0.790	0.871	0.848	0.905	0.907	0.876	0.918	0.855	0.890	1

Supplementary Table 1: Pearson's correlation Matrix and representative scatterplots. Table of Pearson correlation coefficients to all possible pairwise comparisons of replicates in cryo-injury and resection samples.

	MI_1	MI_2	MI_3	MI_5	MI_4	Mean	SD
Total Live Cells	198718	192512	199490	192398	190942		
Total GFP+ cells	19645	13478	18365	7672	19108		
**% GFP+ cells**	9.89	7.00	9.21	3.99	10.01	8.02	2.56
Total Macrophages (F4/80-PE)	2226	1443	2028	983	2675		
Macrophages GFP+	891	599	1160	437	1282		
**% Macrophages GFP+**	40.03	41.51	57.20	44.46	47.93	46.22	6.84
**% Macrophages GFP+ (total GFP+)**	4.54	4.44	6.32	5.70	6.71	**5.54**	1.03
**% Macrophages GFP+ (total cells)**	0.45	0.31	0.58	0.23	0.67	0.45	0.18
Total Fibroblasts (mEF-SK4-APC)	61272	48730	56743	29089	62870		
Fibroblasts GFP+	14992	8781	14635	5772	15345		
**% Fibroblasts GFP+**	24.46795	18.0197	25.79173	19.84255	24.40751	22.51	3.37
**% Fibroblasts GFP+ (total GFP+)**	76.31	65.15	79.69	75.23	80.31	**75.34**	6.09
**% Fibroblasts GFP+ (total cells)**	7.54	4.56	7.34	3.00	8.04	6.10	2.20

Supplementary Table 2: Flow cytometry of relative percentage proportions of GFP^+ macrophages and GFP^+ fibroblasts from Col1α-GFP mice hearts day 7 post-MI (n=5).

Macrophages comprised 5.54% and fibroblasts 75.34% of the total GFP+ cells population. Interestingly macrophages made up only 0.45% of the total cells in the heart, whereas fibroblasts accounted for 6.1%, and of the total macrophage population, 46.2% were GFP^+ compared to 22.5% of fibroblasts.

**sgRNA col4a3bpa 3**	GAAATTAATACGACTCACTATAGGG<br>cagTGGACAAACTACATTCA<br>GTTTTAGAGCTAGAAATAGC
**sgRNA col4a3bpa 4**	GAAATTAATACGACTCACTATAGG<br>GACAGCCTTGCTGAGACAGA<br>GTTTTAGAGCTAGAAATAGC
**sgRNA col4a1 1**	GAAATTAATACGACTCACTATAGGG<br>TTAAATGTATCAGGTTTTCA<br>GTTTTAGAGCTAGAAATAGC
**sgRNA col4a1 4**	GAAATTAATACGACTCACTATAGG<br>GAAGAATGTGATGTTACAAA<br>GTTTTAGAGCTAGAAATAGC
**sgRNA Trio Col4a1 Int23**	GAAATTAATACGACTCACTATAGGG<br>GCATAACAAGGGAATCTACA<br>GTTTTAGAGCTAGAAATAGC

Supplementary Table 3: Oligonucleotides for sgRNA templates.
List of gene-specific oligonucleotides used for sgRNA template, generated with a unique oligonucleotide encoding the T7 RNA polymerase recognition site (in green),the sgRNA target sequence (in red) and an overlap with the tracrRNA (in blue).

**5' col4a3bpasg3**	TGAGGGTTTGAGAGATAAGCGT
**3' col4a3bpasg3**	CCTTCAGCGCAATCCAGC
**5' col4a3bpasg4**	CGCTGAAGGGCAATATTCTCA
**3' col4a3bpasg4**	GCTCTTGACAACCATACAGACA
**5' col4a1 sg1**	TCTTTGTCAGATTGCTCAGTCTT
**3' col4a1 sg1**	ACAGTGCTACAAAATGCTAACCT
**5' col4a1 sg4**	GAAGAGGCTTGTGTTGTTCCAT
**3' col4a1 sg4**	TGTCTCTCACCTTTCTGTCCC
**5' Trio Col4a1 Int23**	TGTTTGATGCACACTTCATGTT
**3' Trio Col4a1 Int23**	CTAAAAAGTTTGGACACCCCTG

Supplementary Table 4: HRMA primers.

Primers were designed to generate a product of approximately 200 base pairs spanning the CRISPR/Cas9 cut site.
