# Supplementary material for: Macrophages directly contribute collagen to scar formation during zebrafish heart regeneration and mouse heart repair
Source: Nat Commun. 2020 Jan 30;11:600. doi: 10.1038/s41467-019-14263-2 (PMC6992796; doi:10.1038/s41467-019-14263-2)
Supplement: Supplementary file 2 — Description of Additional Supplementary Files [file 41467_2019_14263_MOESM2_ESM.docx]

**Description of Additional Supplementary Files:**

**File Name:** Supplementary Data 1

**Description:** Zebrafish Deseq2 differential expression outputs for all the experimental timepoints and conditions studied.

**File Name:** Supplementary Data 2

**Description:** Mouse differential expression outputs for all the experimental timepoints and conditions studied.

**File Name:** Supplementary Data 3

**Description:** Custom R scripts used to generate the cluster overlap significance plot of Supplementary Figure 5. Script1 calculates temporal trend overlaps and p-values-like stem. Script2 displays these graphically as a bubble plot.
