# Supplementary material for: Macrophages directly contribute collagen to scar formation during zebrafish heart regeneration and mouse heart repair
Source: Nat Commun. 2020 Jan 30;11:600. doi: 10.1038/s41467-019-14263-2 (PMC6992796; doi:10.1038/s41467-019-14263-2)
Supplement: Supplementary file 6 — Reporting Summary [file 41467_2019_14263_MOESM6_ESM.pdf]

nature research

Corresponding author(s): Paul Riley

Last updated by author(s): Dec 18, 2019

Reporting Summary

Nature Research wishes to improve the reproducibility of the work that we publish. This form provides structure for consistency and transparency in reporting. For further information on Nature Research policies, see Authors & Referees and the Editorial Policy Checklist .

Statistics

For all statistical analyses, confirm that the following items are present in the figure legend, table legend, main text, or Methods section.

n/a	Confirmed	
☐	☒	The exact sample size (*n*) for each experimental group/condition, given as a discrete number and unit of measurement
☐	☒	A statement on whether measurements were taken from distinct samples or whether the same sample was measured repeatedly
☐	☒	The statistical test(s) used AND whether they are one- or two-sided<br>*Only common tests should be described solely by name; describe more complex techniques in the Methods section.*
☒	☐	A description of all covariates tested
☐	☒	A description of any assumptions or corrections, such as tests of normality and adjustment for multiple comparisons
☐	☒	A full description of the statistical parameters including central tendency (e.g. means) or other basic estimates (e.g. regression coefficient) AND variation (e.g. standard deviation) or associated estimates of uncertainty (e.g. confidence intervals)
☐	☒	For null hypothesis testing, the test statistic (e.g. *F*, *t*, *r*) with confidence intervals, effect sizes, degrees of freedom and *P* value noted<br>*Give P values as exact values whenever suitable.*
☒	☐	For Bayesian analysis, information on the choice of priors and Markov chain Monte Carlo settings
☐	☒	For hierarchical and complex designs, identification of the appropriate level for tests and full reporting of outcomes
☐	☒	Estimates of effect sizes (e.g. Cohen's *d*, Pearson's *r*), indicating how they were calculated

Our web collection on statistics for biologists contains articles on many of the points above.

Software and code

Policy information about availability of computer code

Data collection	Imaging was performed using either an Olympus FV1000 confocal microscope, a Leica DM600 CFS confocal microscope or a Zeiss 780 Upright MP confocal microscope (as described in our methods section).<br>RNA-seq raw sequencing data files (fastq) were downloaded from Illumina Basespace using Basespace module v0.2 to our Computational Bioinformatics Resource Group (CBRG) core server.
Data analysis	Next Generation Sequencing was performed on a NextSeq500 platform using a NextSeqTM500 150 cycle High Output Kit) (Illumina) to generate 80-basepair paired end reads. Read quality was evaluated using FastQC. Zebrafish reads were mapped to the Jul. 2014 Zv10/ danRer10 assembly version of the zebrafish genome using STAR (v.2.4.2a) splice-aware aligner. Count tables were generated using subread FeatureCounts (v1.4.5-p1q), with standard parameters. Differential expression was carried out using DESeq2 R package. Hierarchical clustering was performed on genes significantly differentially expressed in at least one comparison (p-value<0.05). Comparisons where p-values were not available were excluded. log2 Fold Change values from DESeq2 analysis were used to calculate Euclidian distances, and Ward's minimum variance method (Ward D) was used to perform hierarchical clustering (k=9).<br>For mouse, trimmed reads (Trim Galore v.0.3.7, http://www.bioinformatics.babraham.ac.uk/projects/trim_galore, with default settings) were screened for quality (FastQC v.011.3, http://www.bioinformatics.bbsrc.ac.uk/projects/fastqc) and then mapped to the mouse genome (Mm10) using TopHat63 v2.1.10, with Bowtie64 v.2.2.6, using the "b2-sensitive" setting with mate inner distance = 208 and mate standard deviation = 213 determined from test alignments. Transcriptome annotations downloaded from the Illumina iGenomes website (UCSC Mm10, https://support.illumina.com/sequencing/sequencing_software/igenome.html) were used with Cufflinks for transcript mapping. Cuffdiff was used for pair-wise comparisons. Genes with log2 fold change > 2 and FDR < 0.05 were deemed significantly differentially expressed. Cuffdiff results were loaded into R (http://www.R-project.org) for further analysis and plotting, including Principal components analysis (PCA) using the pcaMethods package and heatmap generation with pheatmap (http://CRAN.R-project.org/package=pheatmap). For temporal analyses, raw counts from Cuffdiff were retrieved and genes with < 5 counts in all samples were removed before carrying out tests. DESeq2 was used to identify genes showing differential temporal regulation and STEM v.1.3.8 was used for temporal clustering and gene ontology enrichment analysis of temporal profiles.<br>HMRA Analysis was done on the BioRad Precision Melt Analysis software (v1.3).<br>FACS plots were generated with the FACSDIva 8.0.1 software.

Confocal images were processed using either Zen (Black version), ImageJ or FIJI (NIH Image, Bethesda, MD) softwares.
Graphs and statistical analyses were generated by Graphpad prism, version 7.

For manuscripts utilizing custom algorithms or software that are central to the research but not yet described in published literature, software must be made available to editors/reviewers. We strongly encourage code deposition in a community repository (e.g. GitHub). See the Nature Research guidelines for submitting code & software for further information.

Data

Policy information about availability of data

All manuscripts must include a data availability statement. This statement should provide the following information, where applicable:

- Accession codes, unique identifiers, or web links for publicly available datasets
- A list of figures that have associated raw data
- A description of any restrictions on data availability

Raw and processed data generated in this study were submitted to GEO with the following accession numbers: zebrafish GSE100029 [https://www.ncbi.nlm.nih.gov/geo/query/acc.cgi?acc=GSE100029] and mouse GSE126772 [https://www.ncbi.nlm.nih.gov/geo/query/acc.cgi?acc=GSE126772]. Differential expression outputs are available as Supplementary Data File 1 and Supplementary Data File 2, respectively. Custom R scripts used to generate the cluster overlap significance plot of Supplementary Data Figure 5 are available as Supplementary Data File 3.
Source data for Figures 4-6 and Supplementary Figures 1, 2, 6, 7 and 9 are provided with the paper.

Field-specific reporting

Please select the one below that is the best fit for your research. If you are not sure, read the appropriate sections before making your selection.

[x] Life sciences [ ] Behavioural & social sciences [ ] Ecological, evolutionary & environmental sciences

For a reference copy of the document with all sections, see nature.com/documents/nr-reporting-summary-flat.pdf

Life sciences study design

All studies must disclose on these points even when the disclosure is negative.

Sample size	To ensure the reproducibility and statistical power all experiments were performed in at least triplicates, with a sample size ranging from n=3 to n=9 per replicate.
Data exclusions	No data were excluded from the analyses.
Replication	All experiments were performed at least in 3 independent replicates and the findings were replicated.
Randomization	Samples were not allocated to experimental groups.
Blinding	For zebrafish, scar measurements were done blindly.

Reporting for specific materials, systems and methods

We require information from authors about some types of materials, experimental systems and methods used in many studies. Here, indicate whether each material, system or method listed is relevant to your study. If you are not sure if a list item applies to your research, read the appropriate section before selecting a response.

Materials & experimental systems

n/a	Involved in the study	
[ ]	[x]	Antibodies
[ ]	[x]	Eukaryotic cell lines
[ ]	[ ]	Palaeontology
[ ]	[x]	Animals and other organisms
[ ]	[ ]	Human research participants
[ ]	[ ]	Clinical data

Methods

n/a	Involved in the study	
[ ]	[ ]	ChIP-seq
[ ]	[x]	Flow cytometry
[ ]	[ ]	MRI-based neuroimaging

Antibodies

Antibodies used	Primary antibodies used: anti-CD68 (Abcam, 1:250), anti-Col1a (Abcam, 1:200), Rabbit anti-Collagen I (Abcam ab34710, 1:100), Rat anti-CD68 (Bio-Rad MCA1957, 1:100), Chicken anti-GFP (Abcam ab13970, 1:100), F4/80-PE (BioLegend Inc., 123110, 1:100), anti-mpeg1 (GeneTex, 1:200), MF20 anti-myosin heavy chain (DSHB, 1:200), anti-mCherry (Clontech, 1:200; GeneTex, 1:150). Alexa (405, 488, 555, 594, 633/647) -conjugated secondary antibodies (1:1000, Invitrogen) were used to reveal primary antibody signal.

Validation	All Antibodies were published and validated in previous studies.

Eukaryotic cell lines

Policy information about cell lines

Cell line source(s)	Murine L929 cells were obtained from the American Type Culture Collection (ATCC) and Mouse primary Cardiac microvascular Endothelial Cells (MCEC) were obtained from Cell Biologics.
Authentication	Authentication of L929 cells as described on the ATCC webpage: https://www.lgcstandards-atcc.org/en/Products/All/CCL-1.aspx#generalinformation.<br>Authentication of MCEC as described on the Cell Biologics webpage: http://www.cellbiologics.net/index.php?route=product/product&path=2_47_89_91&product_id=2241
Mycoplasma contamination	We confirm that both L929 and MCEC lines tested negative for mycoplasma contamination.
Commonly misidentified lines<br>(See ICLAC register)	None

Palaeontology

Specimen provenance	N/A
Specimen deposition	N/A
Dating methods	N/A

☐ Tick this box to confirm that the raw and calibrated dates are available in the paper or in Supplementary Information.

Animals and other organisms

Policy information about studies involving animals; ARRIVE guidelines recommended for reporting animal research

Laboratory animals	Wild type, TgBAC(mpeg1:BirA-Citrine)ox122, Tg(βactin:Avi-Cerulean-RanGap)ct700a, TgBAC(mpeg1:BirA-Citrine;βactin:Avi-Cerulean-Rangap)ox133, Gt(foxd3-citrine)ct110, Tg(mpeg1:EGFP)gl22 and Tg(mpeg1:mCherry)gl23 zebrafish were used in this study.<br>Wild-type CD1 mice (Harlan), Col1a2-CreERT2, Col1a1-GFP, R26R-tdTomato, R26R-YFP, hCD68-GFP and GFPtpz-collagen mice were used in this study.
Wild animals	The study did not involve wild animals.
Field-collected samples	The study did not involve samples collected from the field.
Ethics oversight	This study was carried out in accordance to procedures authorized by the UK Home Office in accordance with UK law (Animals Scientific Procedures Act 1986) and approved by the Research Ethics Committee of the University of Oxford.

Note that full information on the approval of the study protocol must also be provided in the manuscript.

Human research participants

Policy information about studies involving human research participants

Population characteristics	N/A
Recruitment	N/A
Ethics oversight	N/A

Note that full information on the approval of the study protocol must also be provided in the manuscript.

Clinical data

Policy information about clinical studies

All manuscripts should comply with the ICMJEguidelines for publication of clinical research and a completedCONSORT checklist must be included with all submissions.

Clinical trial registration	N/A
Study protocol	N/A

Data collection	N/A
Outcomes	N/A

ChIP-seq

Data deposition

☐ Confirm that both raw and final processed data have been deposited in a public database such as GEO.

☐ Confirm that you have deposited or provided access to graph files (e.g. BED files) for the called peaks.

Data access links<br>*May remain private before publication.*	N/A
Files in database submission	N/A
Genome browser session<br>(e.g. UCSC)	N/A

Methodology

Replicates	N/A
Sequencing depth	N/A
Antibodies	N/A
Peak calling parameters	N/A
Data quality	N/A
Software	N/A

Flow Cytometry

Plots

Confirm that:

☒ The axis labels state the marker and fluorochrome used (e.g. CD4-FITC).

☒ The axis scales are clearly visible. Include numbers along axes only for bottom left plot of group (a 'group' is an analysis of identical markers).

☒ All plots are contour plots with outliers or pseudocolor plots.

☒ A numerical value for number of cells or percentage (with statistics) is provided.

Methodology

Sample preparation	Mouse single cell cardiac tissue suspensions were prepared by mincing hearts and gentle agitation in collagenase 500 units per ml in HBSS solution for 1 hour at 37°C. Samples were passed through a 70µm filter, washed and resuspended in 1% FBS/PBS. Samples were incubated with FcR-block, and then labelled with anti-CD45 (Biolegend 103124, 1:200), anti-CD11b (Biolegend 101206, 1:100), anti-Ly6G (Biolegend 127636, 1:200), anti-F4/80 (Biolegend 123110, 1:100), anti-Ly6C (Biolegend 128026 1:800), anti-CD206 (Biolegend 141721, 1:100).<br>Zebrafish GFP+ macrophages were isolated from Tg(mpeg1:EGFP)gl22 operated adult hearts or whole embryos by FACS. mCherry+ macrophages were isolated from Tg(mpeg1:mCherry)gl23 "trio"-tagged whole embryos by FACS. Neural crest cells used as control in adoptive cell transfer experiment were isolated from 16-somite stage Gt(foxd3-citrine)ct110 embryos. Prior to FACS, tissue was dissociated using 20 mg/ml collagenase in 0.05% Trypsin/0.53 mM EDTA/1xHBSS buffer to obtain single cell suspensions. Reaction was stopped in 10 mM HEPES/0.25% BSA/1xHBSS buffer and GFP+ and/or mCherry+ cells were sorted.
Instrument	FACSAriaIII, BD Biosciences Fusion System.
Software	Analysis was performed using FlowJo v10.0.8.
Cell population abundance	The purity of the post-sort fractions was measured ad hoc by the core flow cytometry facility and found to be in excess of 98% pure.
Gating strategy	Cell populations were gated as shown in Supplementary Data Figure 2 and Supplementary Data Figure 8. Briefly, doublets were excluded (by FSC-W vs FSC-A) and dead cells removed by 7-AAD. Myeloid cells were gated for CD45+, CD11b+, and neutrophils identified by positivity for Ly6G. Macrophages were identified as Ly6G F4/80+ cells. Monocytes were identified as Ly6G F4/80+ LyChi/lo cells.

Zebrafish cells were first gated by size and granularity, doublets and then by fluorescent markers. WT-derived (fluorescence-negative) cells were used as an initial gate to remove any autofluorescent cells from our experiments (as shown in Supplementary Data Figure 8).

☒ Tick this box to confirm that a figure exemplifying the gating strategy is provided in the Supplementary Information.

Magnetic resonance imaging

Experimental design

Design type	N/A
Design specifications	N/A
Behavioral performance measures	N/A

Acquisition

Imaging type(s)	N/A
Field strength	N/A
Sequence & imaging parameters	N/A
Area of acquisition	N/A

Diffusion MRI ☐ Used ☒ Not used

Preprocessing

Preprocessing software	N/A
Normalization	N/A
Normalization template	N/A
Noise and artifact removal	N/A
Volume censoring	N/A

Statistical modeling & inference

Model type and settings	N/A
Effect(s) tested	N/A

Specify type of analysis: ☐ Whole brain ☐ ROI-based ☐ Both

Statistic type for inference (See Eklund et al. 2016)	N/A
Correction	N/A

Models & analysis

n/a	Involved in the study
☒	☐ Functional and/or effective connectivity
☒	☐ Graph analysis
☒	☐ Multivariate modeling or predictive analysis
